# Exploring body weight-influencing gut microbiota by elucidating the association with diet and host gene expression

**DOI:** 10.1038/s41598-023-32411-z

**Published:** 2023-04-05

**Authors:** Shino Nemoto, Tetsuya Kubota, Hiroshi Ohno

**Affiliations:** 1 Laboratory for Intestinal Ecosystem, RIKEN Center for Integrative Medical Sciences, Kanagawa, Japan; 2 Division of Diabetes and Metabolism, The Institute of Medical Science, Asahi Life Foundation, Tokyo, Japan; 3 Department of Clinical Nutrition, National Institutes of Biomedical Innovation, Health and Nutrition, Tokyo, Japan; 4 Division of Cardiovascular Medicine, Toho University Ohashi Medical Center, Tokyo, Japan; 5 Department of Diabetes and Metabolic Diseases, Graduate School of Medicine, The University of Tokyo, Tokyo, Japan; 6 Laboratory for Immune Regulation, Graduate School of Medical and Pharmaceutical Sciences, Chiba University, Chiba, Japan; 7 Immunobiology Laboratory, Graduate School of Medical Life Science, Yokohama City University, Kanagawa, Japan

**Keywords:** Microbiology, Molecular biology, Physiology

## Abstract

We aimed to identify gut microbiota that influences body weight by elucidating the association with diets and host genes. Germ-free (GF) mice with and without fecal microbiota transplant (FMT) were fed a normal, high-carbohydrate, or high-fat diet. FMT mice exhibited greater total body weight; adipose tissue and liver weights; blood glucose, insulin, and total cholesterol levels; and oil droplet size than the GF mice, regardless of diet. However, the extent of weight gain and metabolic parameter levels associated with gut microbiota depended on the nutrients ingested. For example, a disaccharide- or polysaccharide-rich diet caused more weight gain than a monosaccharide-rich diet. An unsaturated fatty acid-rich diet had a greater microbial insulin-increasing effect than a saturated fatty acid-rich diet. Perhaps the difference in microbial metabolites produced from substances taken up by the host created metabolic differences. Therefore, we analyzed such dietary influences on gut microbiota, differentially expressed genes between GF and FMT mice, and metabolic factors, including body weight. The results revealed a correlation between increased weight gain, a fat-rich diet, increased *Ruminococcaceae* abundance, and decreased claudin 22 gene expression. These findings suggest that weight regulation might be possible through the manipulation of the gut microbiota metabolism using the host’s diet.

## Introduction

Progress in gut microbiota research over the past decade has provided evidence of a causal relationship between microbiota and obesity^1–9^, emphasizing the need for identifying target gut microorganisms to prevent and treat the disease^10–12^. However, the extent of their influence remains unknown, and additional research is required to better understand the mechanisms underlying the relationship between gut microbiota and host metabolism.

Commonly used approaches to manipulate the gut microbiota involve indirect disruption (by altering the host diet or supplying probiotics, prebiotics, or antibiotics) or direct inoculation of the microbiota (via the fecal microbiota transplantation method)^13^. Turnbaugh’s study^14^ on the involvement of gut microbiota in obesity via direct colonization is well known, showing that germ-free (GF) mice directly transplanted with feces obtained from obese mice (with high amounts of "obesogenic microorganisms") are considerably fatter than mice transplanted with feces obtained from lean mice. Accordingly, a comparative analysis of the gut microbiota of obese and lean mice can help identify the gut microbiota involved in obesity.

Thus, we monitored the weight gained by GF mice administered feces from obese and non-obese mice; however, we did not observe any accelerated progression of obesity in the former. Even though the host environment was altered by low- and high-fat diets (Supplementary Fig. S1), we found no evidence of the presence of the “obesogenic microorganisms” in feces from fecal-administered mice. Nevertheless, the mice that received the fecal microorganisms gained more weight than the GF mice (Supplementary Fig. S1), clearly indicating the presence of microorganisms that contribute to host weight gain. Therefore, we changed the method for disrupting gut microbiota. Considering that both the body weight (BW) of the host and composition of gut microbiota are greatly influenced by diet^15,16^, disrupting the gut microbiota indirectly by manipulating the nutritional environment of the host may be more effective. Specifically, a comparative analysis of the altered gut microbiota and BW in response to the different nutrients will allow for the identification of obesogenic microorganisms.

Recent studies have shown that disruptions in the gut environment caused by changes in the nutritional status of the host can affect host gene expression in a wide range of tissues, leading to altered metabolism^17–19^. Therefore, analysis of changes in host gene expression along with the abundance of gut microbiota in response to nutritional status will help elucidate the molecular mechanisms by which the microbiota-host interaction affects obesity.

We identified gut microorganisms involved in obesity by feeding various diets to GF mice with and without fecal microbiota transplant (FMT), and examining the BW, tissue weight, blood metabolic factors, and differentially expressed genes (DEGs) in each tissue. Furthermore, we discussed the associated mechanisms between the identified microbiota and obesity from the viewpoint of the "nutrient-gut microbiota-host gene" linkage.

## Results

### Body weight and microbiota

**Figure 1:**
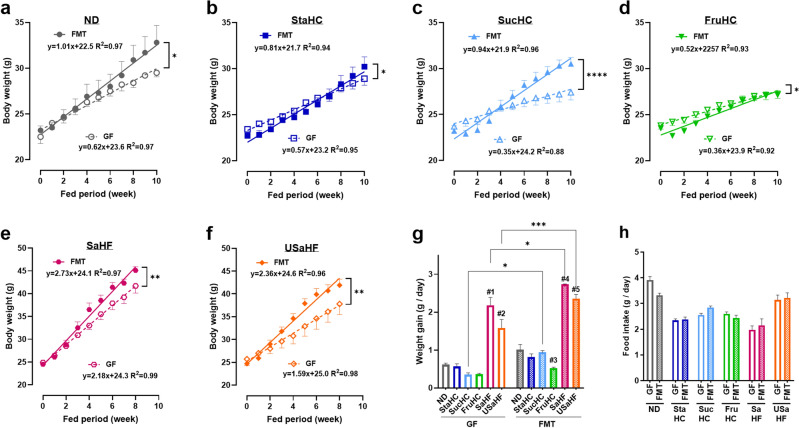
Difference in body weight between germ-free (GF) and fecal microbiota transplant (FMT) mice fed six different diets. The diets included (**a**) ND (n = 4), (**b**) StaHC (n = 7), (**c**) SucHC (n = 4), (**d**) FruHC (n = 7), (**e**) SaHF (n = 4), and (**f**) USaHF (n = 4). Values are presented as the mean ± SEM. Linear regression lines were compared using a two-tailed analysis of covariance. (**g**) Amount of weight gain/day. (**h**) Amount of food intake/d. Multiple comparisons were analyzed using a two-way analysis of variance with the Bonferroni post-hoc test. Asterisks (*) indicate significant differences between GF and FMT (*p < 0.05, **p < 0.01, ***p < 0.001, and ****p < 0.0001). The # denotes the following significant differences between the diet groups: #1 *p* < 0.0001, SaHF vs ND, StaHC, SucHC, and FruHC; #2 *p* < 0.0001, USaHF vs ND, StaHC, SucHC, and FruHC; *p* < 0.01, USaHF vs SaHF; #3 *p* = 0.016, FruHC vs ND; #4 *p* < 0.0001, SaHF vs ND, StaHC, SucHC, and FruHC; #5 *p* < 0.0001, USaHF vs ND, StaHC, SucHC, and FruHC. *ND* normal diet, *StaHC* high-starch diet, *SucHC* high-sucrose diet, *FruHC* high-fructose diet, *SaHF* saturated fatty acid-rich high-fat diet, *USaHF* unsaturated fatty acid-rich high-fat diet, *SEM* standard error of the mean.

To examine the association of gut microbiota with host BW on the basis of diet, we prepared FMT mice that were administered feces from standard chow diet-fed mice and compared them with GF mice. The mice were fed a normal diet (ND), any of the three high-carbohydrate (HC) diets (high-starch diet, StaHC; high-sucrose diet, SucHC; high-fructose diet, FruHC), and either of two high-fat (HF) diets (saturated fatty acid-rich HF diet, SaHF; and unsaturated fatty acid-rich HF diet, USaHF) for 8–10 weeks. The time course of BW for each diet is plotted in Fig. 1a–f. The regression lines with age as a covariate were compared using analysis of covariance. In all diets, FMT mice that contained microorganisms gained significantly more weight (ND: *p* = 0.014, StaHC: *p* = 0.020, SucHC: *p* < 0.0001, FruHC: *p* = 0.038, SaHF: *p* = 0.002, and USaHF: *p* = 0.002) than GF mice. A comparison of BW gain/day (increasing ratio) among the diets is shown in Fig. 1g. The ratio of weight gain in FMT mice in the FruHC group was significantly lower than that in the ND group (*p* = 0.016), which may be due in part to the lower food intake (Fig. 1h). Food intake did not differ between GF and FMT mice, regardless of the diet (Fig. 1h).

### Tissue weight and microbiota

**Figure 2:**
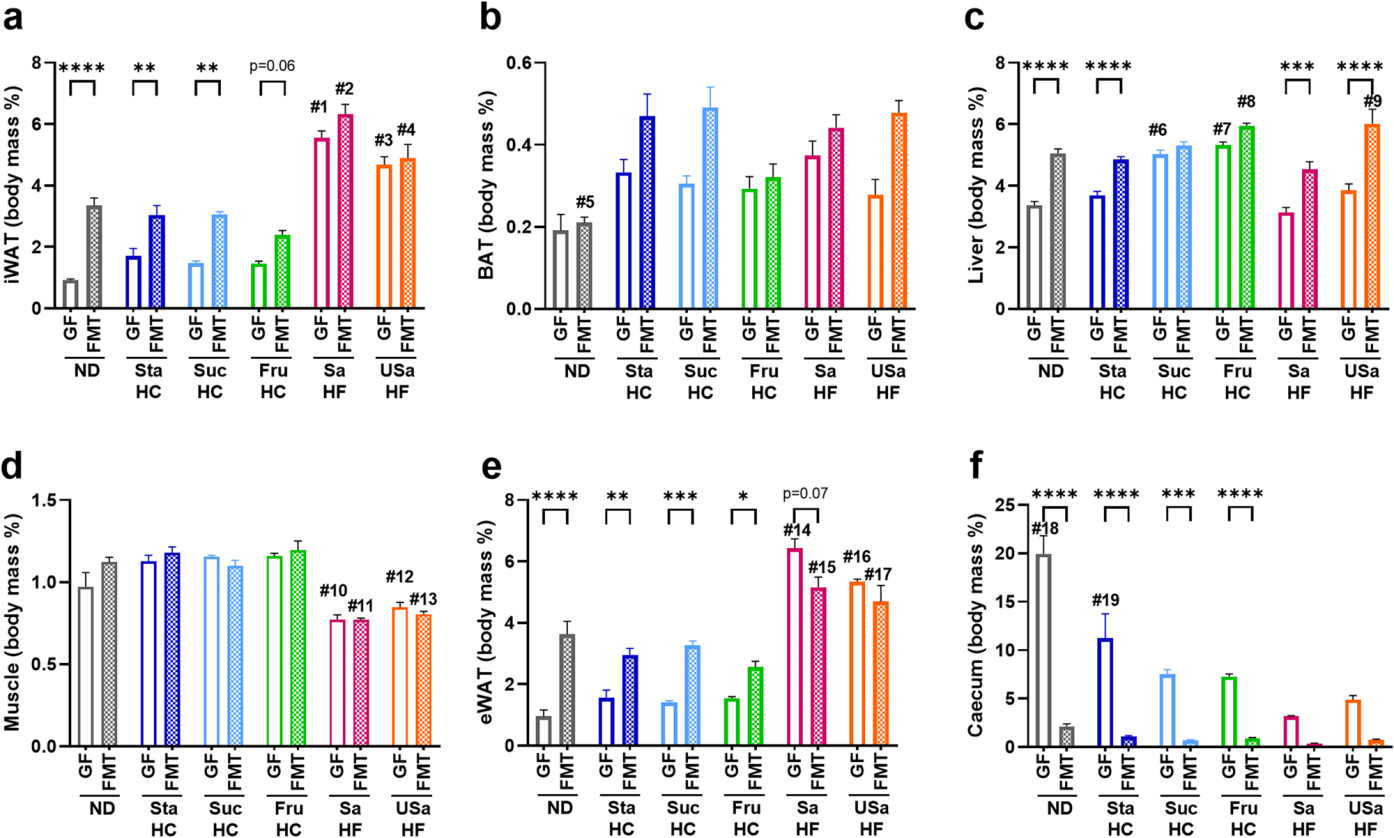
Difference in tissue weights between germ-free (GF) and fecal microbiota transplant (FMT) mice fed six different diets. The tissues analyzed included: (**a**) iWAT, (**b**) BAT, (**c**) liver (**d**) muscle, (**e**) eWAT, and (**f**) caecum with content. Values are expressed as the relative weight calculated based on organ weight/body weight (BW) (body mass %) and presented as the mean + SEM (n = 4 or 7). Multiple comparisons were analyzed using a two-way analysis of variance, followed by Tukey’s test. Asterisks (*) indicate significant differences between GF and FMT mice (*p < 0.05, **p < 0.01, ***p < 0.001, and ****p < 0.0001). The # denotes the significance between the diet groups, and the statistical results pertaining to the number of # correspond to the number of # marked in Supplementary Table S3. *iWAT* inguinal white adipose tissue, *BAT* brown adipose tissue, *muscle* skeletal muscle, *eWAT* epididymal white adipose tissue.

Figure 2 shows a comparison of relative tissue weight (% of BW) between GF and FMT mice at 18–20 weeks of age after 8–10 weeks on different diets. Overall, the tissue weight of inguinal white adipose tissue (iWAT, Fig. 2a), brown adipose tissue (BAT, Fig. 2b), and liver (Fig. 2c) in FMT mice tended to be greater than that in GF mice. The effects of microorganisms on tissue weight across the diets were analyzed using two-way ANOVA, with microorganisms representing intra-subject variability and diets representing inter-subject variability. Particularly, the weight of iWAT and epididymal white adipose tissue (eWAT) in ND (*p* < 0.0001 for iWAT and eWAT) and HC (*p* < 0.01 for iWAT and eWAT in StaHC and SucHC, *p* = 0.06 for iWAT and* p* < 0.05 for eWAT in FruHC) diet groups (Fig. 2a,e) and that of the liver in ND (*p* < 0.0001) and HF (*p* < 0.001 in SaHF and *p* < 0.0001 in USaHF) diet groups (Fig. 2c) were significantly higher for FMT mice than for GF mice. Notably, the eWAT of FMT mice in HF diet groups was smaller in size than that of GF mice (Fig. 2e). The caecum of FMT mice was considerably smaller in size than that of GF mice in all diet groups (Fig. 2f). Absolute weight comparisons between GF and FMT mice were similar to relative weight comparisons (Supplementary Fig. S2).

### Oil droplet staining and microbiota

**Figure 3:**
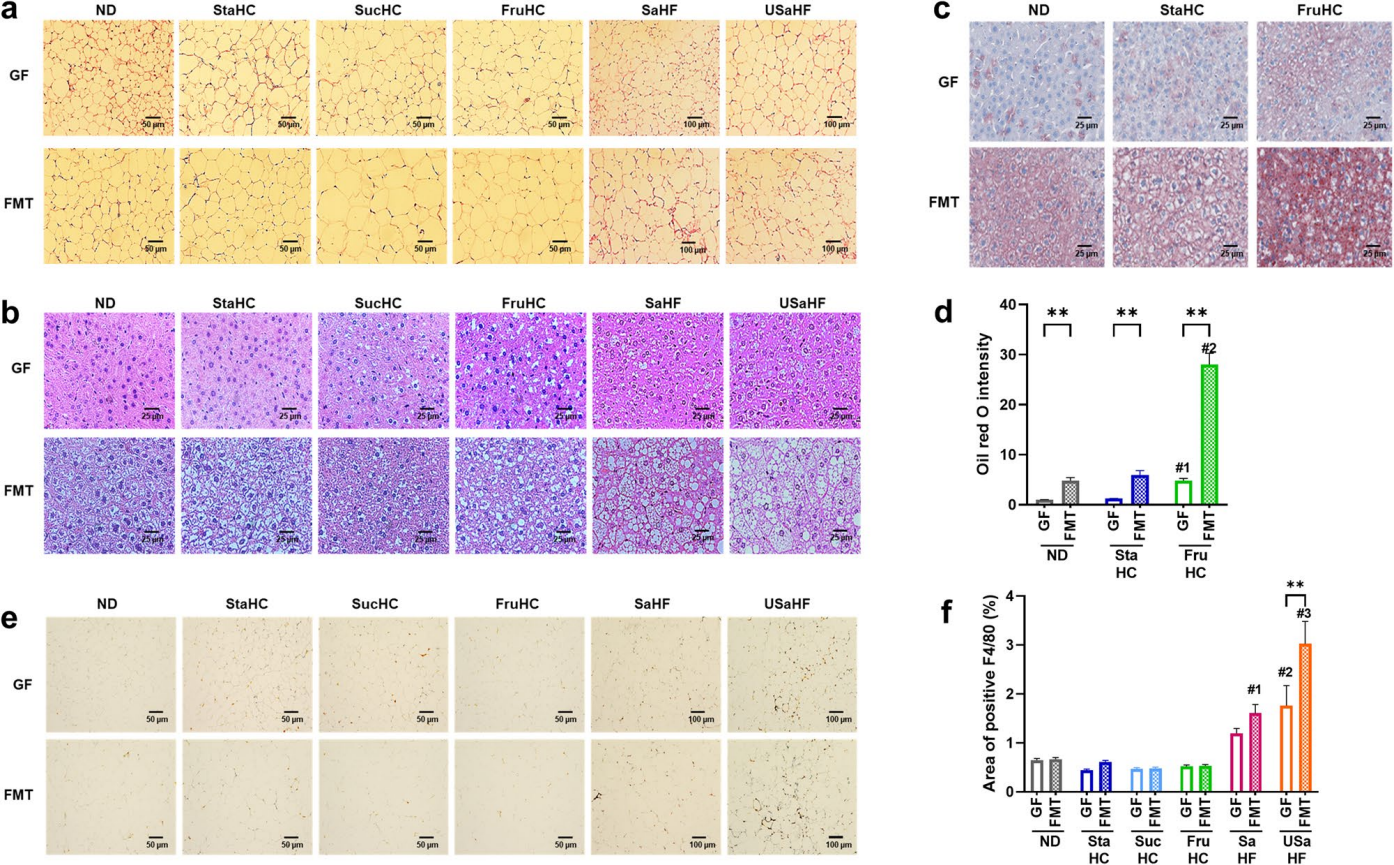
Histological analysis of iWAT and liver in germ-free (GF) and fecal microbiota transplant (FMT) mice fed six different diets. (**a**) Representative images of iWAT stained with hematoxylin and eosin (HE). Scale bar: 50 μm for ND, StaHC, SucHC, and FruHC; 100 μm for SaHF and USaHF. (**b**) Representative images of the liver stained with HE. Scale bar, 20 μm. (**c**) Representative images of the liver stained with Oil Red O. Scale bar, 20 μm. (**d**) Quantification of Oil Red O positive area. Values are presented as the mean + SEM (n = 4 per group); the GF-ND group was calculated as one. Multiple comparisons were analyzed using a two-way analysis of variance, followed by Tukey’s test. Asterisks (*) indicate significant differences between GF and FMT mice (**p < 0.01), and the # denotes the following significances between the diet groups: #1 FruHC vs ND (*p* = 0.037) and StaHC (*p* = 0.044); #2 FruHC vs ND and StaHC (*p* < 0.0001). (**e**) Immunohistochemistry for F4/80 staining of iWAT. Scale bar, 50 μm for ND, StaHC, SucHC, and FruHC; 100 μm for SaHF and USaHF. Magnification, × 200. (**f**) Quantification of the F4/80 positive area. Values are presented as the mean + SEM (n = 4 per group). Multiple comparisons were analyzed using a two-way analysis of variance, followed by Tukey’s test. Asterisks (*) indicate significant differences between GF and FMT (**p < 0.01), and the # denotes the following significances between the diet groups: #1 SaHF vs ND (*p* = 0.043), StaHC (*p* = 0.026), SucHC (*p* = 0.007), and FruHC (*p* = 0.012); #2 USaHF vs ND (*p* = 0.008), StaHC (*p* = 0.001), SucHC (*p* = 0.001), and FruHC (*p* = 0.002); #3 USaHF vs ND, StaHC, SucHC, and FruHC (*p* < 0.0001), vs SaHF (*p* = 0.0003). *iWAT* inguinal white adipose tissue, *ND* normal diet, *StaHC* high-starch diet, *SucHC* high-sucrose diet, *FruHC* high-fructose diet, *SaHF* saturated fatty acid-rich high-fat diet, *USaHF* unsaturated fatty acid-rich high-fat diet.

As the weights of white adipose tissues and liver were greatly affected by the presence of gut microorganisms (Fig. 2), we investigated the morphological characteristics of iWAT and liver (Fig. 3). Hematoxylin and eosin (H&E) staining of iWAT showed that adipocytes in FMT mice were larger than those in GF mice regardless of diet (Fig. 3a). In addition, H&E staining of the liver showed that the lipid droplet-like areas that appeared in FMT mice were greater in number than those in GF mice (Fig. 3b). Among FMT mice, these lipid droplet-like areas in high-fat diet groups (SaHF and USaHF) were larger than those in ND and HC diet groups, and the area in USaHF group was larger than that in SaHF (Fig. 3b). Notably, such lipid droplet-like areas were as abundant in the liver of GF mice in the FruHC diet group as in the liver of FMT mice (Fig. 3b). To confirm these areas were lipid droplets, we performed Oil Red O staining (Fig. 3c,d). The livers of FMT mice were stained to a greater extent than the livers of GF mice, and the livers of mice in the FruHC diet group were stained to a greater extent than those in the ND and StaHC diet groups (Fig. 3c,d). These results indicate that gut microorganisms promote adipocyte hypertrophy in white adipose tissues and fat deposition in the liver. Furthermore, to investigate the association of gut microorganisms with inflammation, which is induced by fat and considered one of the causes of obesity, we performed immunohistochemistry for the macrophage marker F4/80 present in iWAT sections (Fig. 3e,f). A greater F4/80 expression region was observed in sections of FMT mice in the USaHF diet group than that in GF mice, while no difference was observed between GF and FMT mice in other diet groups (Fig. 3e,f). In addition, the section of the USaHF group in both GF and FMT mice showed greater F4/80 expression than other diet groups (Fig. 3e,f).

### Effects of microbiota on blood components involved in metabolism

**Figure 4:**
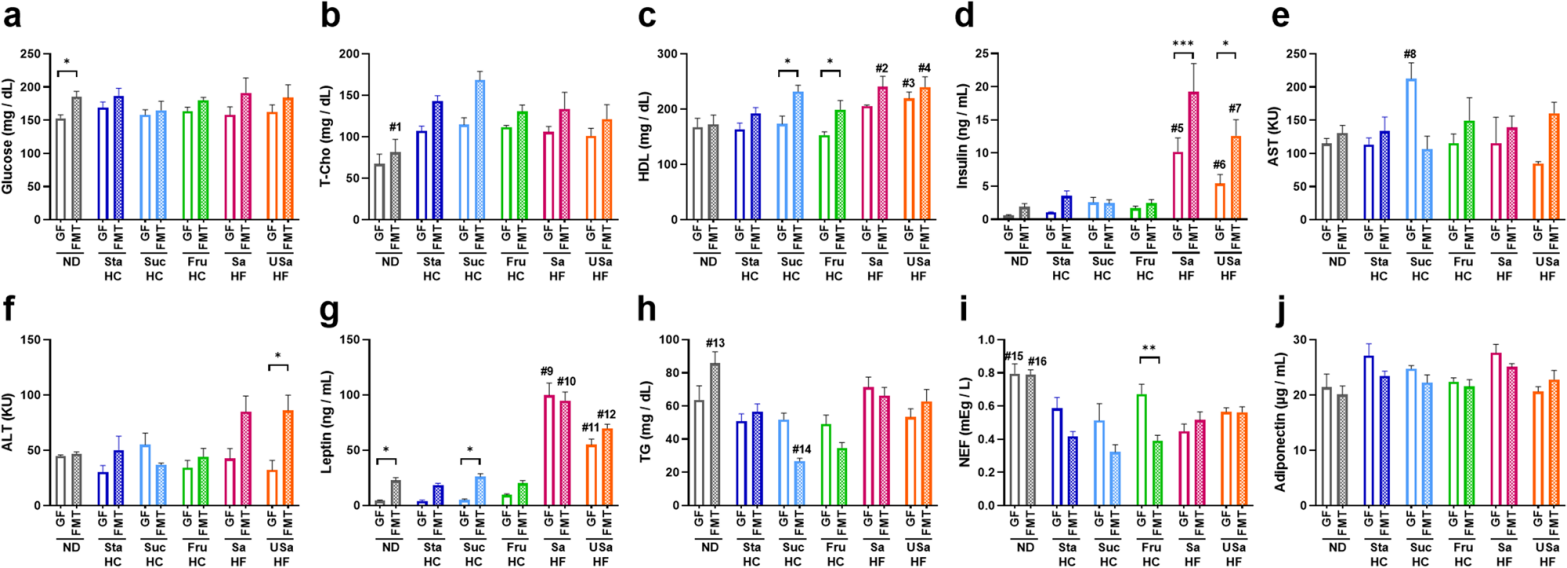
Differences in metabolic parameters of the plasma between germ-free (GF) and fecal microbiota-transplant (FMT) mice fed six different diets. (**a**) Glucose, (**b**) T-Cho, (**c**) HDL, (**d**) insulin, (**e**) AST, (**f**) ALT, (**g**) leptin, (**h**) TG, (**i**) NEF, and (**j**) adiponectin. Values are presented as the mean + SEM (n = 4–7). Asterisks (*) indicate significant differences between GF and FMT (*p < 0.05, **p < 0.01, and ***p < 0.001), and the # denotes the following significances between the diet groups by two-way analysis of variance, followed by Tukey’s test: #1 ND vs StaHC (*p* = 0.001), vs SucHC (*p* < 0.0001), vs FruHC (*p* = 0.019); #2 ND vs SaHF (*p* = 0.031); #3 FruHC vs USaHF (*p* = 0.013); #4 ND vs USaHF (*p* = 0.034); #5 vs SaHF vs ND (*p* < 0.0001), vs StaHC (*p* = 0.0001), vs SucHC (*p* = 0.009), vs FruHC (*p* = 0.0003); #6 SaHF vs USaHF (*p* = 0.042), vs ND, StaHC, SucHC, and FruHC (*p* < 0.0001); #7 USaHF vs SaHF (*p* = 0.042), vs ND, StaHC, SucHC, and FruHC (*p* < 0.0001); #8 StaHC vs USaHF (*p* = 0.033); #9 SaHF vs USaHF, ND, StaHC, SucHC, and FruHC (*p* < 0.0001); #10 SaHF vs USaHF (*p* = 0.006), vs ND, StaHC, SucHC, and FruHC (*p* < 0.0001); #11 USaHF vs SaHF, ND, StaHC, SucHC, and FruHC (*p* < 0.0001); #12 USaHF vs SaHF (*p* = 0.006), vs ND, StaHC, SucHC, and FruHC (*p* < 0.0001); #13 ND vs StaHC (*p* = 0.027), vs SucHC, FruHC (*p* < 0.0001); #14 SucHC vs SaHF (*p* = 0.045); #15 ND vs SucHC (*p* = 0.024), vs SaHF (*p* = 0.002); #16 ND vs SaHF (*p* = 0.031), vs StaHC, SucHC, and FruHC (*p* < 0.0001). *T-Cho* total cholesterol, *HDL* high-density lipoprotein cholesterol, *AST* aspartate aminotransferase, *ALT* alanine aminotransferase, *TG* triglyceride, *NEF* non-esterified fatty acids, *ND* normal diet, *StaHC* high-starch diet, *SucHC* high-sucrose diet, *FruHC* high-fructose diet, *SaHF* saturated fatty acid-rich high-fat diet, *USaHF* unsaturated fatty acid-rich high-fat diet.

Assessment of the blood components involved in metabolism revealed a few major trends that differed between GF and FMT mice (Fig. 4): the common being regardless of the diet is that the concentrations of glucose (Fig. 4a), total cholesterol (T-Cho, Fig. 4b), and high-density lipoprotein cholesterol (HDL, Fig. 4c) were higher in FMT mice than in GF mice, while insulin (Fig. 4d), aspartate aminotransferase (AST, Fig. 4e), and alanine aminotransferase (ALT, Fig. 4f) levels were lower in GF mice than in FMT mice in most diet groups except SucHC. Particularly, insulin levels in FMT mice of HF diet groups were significantly higher (*p* < 0.001 in SaHF and *p* < 0.05 in USaHF) and approximately twice that of the levels in GF mice (Fig. 4d). Notably, leptin levels in the GF mice of HF diet groups were as high as those in FMT mice, while those in GF mice of ND and HC diet groups were considerably lower than those in FMT mice (Fig. 4g). Triglyceride (TG, Fig. 4h) and non-esterified fatty acid (NEF, Fig. 4i) levels were also unaffected in the HF group; however, they were decreased by gut microorganisms in the SucHC and FruHC groups. Adiponectin levels showed no difference (Fig. 4j).

### Host genes affected by gut microbiota

To identify host genes affected by gut microorganisms, we analyzed DEGs between GF and FMT mice in various tissues (iWAT, eWAT, BAT, muscle, liver, duodenum, and ileum) in response to diet (ND, StaHC, SucHC, FruHC, SaHF, and USaHF). Table 1 shows that the number of DEGs obtained (*p* < 0.05, |log2FC| > 0) in the duodenum was higher than that in the muscle, BAT, and iWat (*p* = 0.06, 0.07, and 0.15, respectively; two-way analysis of variance [two-way ANOVA], followed by Tukey’s test), suggesting that the duodenum was affected most by gut microorganisms. Mice in the ND diet group had relatively more DEGs, while those in the FruHC diet group had few (ND vs FruHC, *p* = 0.004; ND vs SaHF, *p* = 0.009, two-way ANOVA, followed by Tukey's test), indicating that the gut microbiota was strongly affected by ND diet and weakly so by the FruHC diet. Of the DEGs obtained, 27 in liver, 15 in duodenum, 13 in eWAT, four in ileum, three in iWAT, three in BAT, and one gene in muscle were common across all diet groups for any given tissue (Table 1). However, none of the DEGs were common across tissues for any diet group (Table 1). The 66 DEGs obtained are presented in Table 2, and the gene expression levels (GELs) in both GF and FMT mice based on diet are shown in Supplementary Fig. S3. To identify host genes associated with gut microorganism-mediated weight regulation, we analyzed the fluctuations in GELs related to gut microorganisms (Supplementary Fig. S3) and BW (Fig. 1) that varied with dietary components. The magnitude of BW affected by gut microorganisms was calculated by subtracting the BW of GF mice from that of FMT mice and expressed in two ways; the increased absolute weight (BW on week 8 − baseline BW [week 0]) was described as the absolute weight (g) ([BW (g) FMT − BW (g) GF] in Table 2) and the increasing ratio (where “s” stands for slope) was described as ([BW (s) FMT − BW (s) GF] in Table 2). The magnitude of GEL affected by gut microorganisms was calculated by subtracting the GEL of GF mice from that of FMT mice (described as [GEL (au) FMT − GEL (au) GF] in Table 2, where “au” stands for arbitrary unit). Subsequently, the correlation coefficients between the two magnitudes were calculated (Pearson's r and *p*-values in Table 2), and genes with Pearson's r > |0.7| (in bold) were designated as host genes associated with gut microorganism-mediated weight regulation (17 genes in bold letters, Table 2).

**Table 1:** Number of differentially expressed genes (DEGs) between germ-free mice and fecal microbiota-transplant mice fed six different diets.

	ND	StaHC	SucHC	FruHC	SaHF	USaHF	6 diets
iWAT	4162	2626	3170	863	192	1234	3
eWAT	5501	1888	3565	1528	3116	1468	13
BAT	3045	1570	2072	938	2321	926	3
Muscle	1994	925	3735	534	1309	2187	1
Liver	4039	2516	3617	2222	1486	4192	27
Duodenum	4741	4597	1878	5073	3134	3277	15
Ileum	5754	2478	1523	820	1602	4557	4
7 tissues	0	0	0	0	0	0	0

DEGs in seven tissues were screened using the criteria of *p* < 0.05 and fold change > 1.

Six diets, the number of DEGs common across all diet groups; seven tissues, the number of DEGs common across all tissues within a diet group; iWAT, inguinal white adipose tissue; eWAT, epididymal white adipose tissue; BAT, brown adipose tissue; muscle, skeletal muscle; ND, normal diet; StaHC, high-starch diet; SucHC, high-sucrose diet; FruHC, high-fructose diet; SaHF, saturated fatty acid-rich high-fat die; USaHF, unsaturated fatty acid-rich high-fat diet.

**Table 2:** Host genes associated with gut microorganism-mediated weight regulation.

	[GEL(au)FMT − GEL(au)GF] vs			
	[BW(g)FMT − BW(g)GF]		[BW(s)FMT − BW(s)GF]	
	r	*p*	r	*p*
eWAT				
*** Cldn22***	**0.83**	**0.04**	**0.72**	**0.11**
*** Prkag3***	**0.81**	**0.05**	**0.65**	**0.17**
*** Scpep1***	**0.74**	**0.09**	**0.59**	**0.22**
*** Srpx2***	**0.71**	**0.11**	**0.53**	**0.27**
*** Mfsd7a***	**0.70**	**0.12**	**0.56**	**0.25**
* Sfrp5*	0.69	0.13	0.54	0.27
* Duoxa1*	0.66	0.15	0.52	0.29
* Zdhhc2*	0.65	0.16	0.51	0.30
* B430212C06Rik*	0.65	0.17	0.53	0.28
* Syp*	0.64	0.17	0.46	0.35
* Tmem45b*	0.64	0.17	0.51	0.30
* Aldh9a1*	0.62	0.19	0.51	0.30
* Dap*	0.23	0.66	0.12	0.83
iWAT				
* Cpne2*	− 0.35	0.49	0.33	0.52
* Pus10*	− 0.30	0.56	− 0.30	0.56
* Gabrr2*	− 0.24	0.65	− 0.29	0.58
BAT				
*** Lox***	**0.96**	**< 0.01**	**0.92**	**0.01**
*** Tgm2***	**0.91**	**0.01**	**0.84**	**0.03**
* Gpx8*	0.42	0.40	0.23	0.66
Liver				
*** Or7d12-ps1***	− **0.96**	**< 0.01**	− **0.95**	**< 0.01**
*** Cryl1***	**0.85**	**0.03**	**0.90**	**0.01**
*** Pepd***	**0.79**	**0.06**	**0.85**	**0.03**
*** Cblc***	**0.77**	**0.07**	**0.69**	**0.13**
*** Apoa4***	**0.68**	**0.14**	**0.70**	**0.12**
* Wfdc2*	0.45	0.37	0.36	0.49
* Sephs2*	0.38	0.46	0.47	0.35
* Pyroxd2*	0.32	0.54	0.12	0.82
* Pyroxd2*	0.32	0.54	0.12	0.82
* Ephx1*	0.31	0.54	0.23	0.67
* Tex2*	− 0.27	0.60	− 0.44	0.38
* Coq8a*	− 0.26	0.61	− 0.31	0.55
* Unc119*	0.26	0.62	0.04	0.93
* Plin2*	0.23	0.66	0.05	0.93
* L2hgdh*	0.23	0.67	0.13	0.80
* Nars2*	0.19	0.71	0.21	0.68
* Atp9a*	0.19	0.72	0.01	0.98
* Acp5*	0.19	0.72	0.33	0.53
* Serinc2*	− 0.10	0.85	− 0.26	0.61
* Cyp3a44*	0.09	0.86	− 0.07	0.90
* Cyp3a11*	0.09	0.87	− 0.07	0.89
* P2ry1*	− 0.08	0.88	− 0.18	0.74
* Cyp3a41b*	0.08	0.88	− 0.08	0.88
* Serpina6*	0.08	0.88	0.00	1.00
* Cyp3a41a*	0.08	0.89	− 0.08	0.88
* Cyp3a16*	0.06	0.92	− 0.10	0.85
* Slc35f2*	0.04	0.94	− 0.16	0.76
Muscle				
* Tmed10*	− 0.30	0.56	− 0.41	0.42
Duodenum				
*** Nlrc5***	**0.90**	**0.02**	**0.78**	**0.07**
*** Tnk1***	− **0.86**	**0.03**	− **0.84**	**0.03**
*** Wfs1***	**0.81**	**0.05**	**0.83**	**0.04**
*** Gm5475***	− **0.75**	**0.09**	− **0.88**	**0.02**
* St6gal1*	− 0.68	0.14	− 0.46	0.36
* Pccb*	0.53	0.28	0.54	0.27
*** Slc11a2***	− **0.52**	**0.28**	− **0.73**	**0.10**
* Wdr83*	0.51	0.30	0.47	0.34
* Lax1*	− 0.49	0.32	− 0.30	0.56
* Prkacb*	0.31	0.54	0.28	0.59
* Fyn*	0.27	0.60	0.17	0.75
* Stk3*	0.17	0.75	0.08	0.88
* Cyba*	0.15	0.78	− 0.06	0.92
* Cyp4b1-ps2*	− 0.14	0.79	− 0.16	0.76
* Tbc1d31*	0.10	0.85	− 0.15	0.77
Ileum				
* Ccdc146*	− 0.48	0.33	− 0.37	0.47
* Tcf23*	− 0.30	0.56	− 0.23	0.66
* 1700016C15Rik*	0.04	0.93	0.25	0.64
* Irf1*	0.04	0.94	− 0.10	0.85

The correlation between differentially expressed genes (DEGs) common across six different diet groups between germ-free mice (GF) and fecal microbiota-administered mice (FMT) and body weight (BW) is expressed by the Pearson’s correlation coefficient (r) and associated *p*-value (*p*). The genes in bold text are with |r| > 0.7.

iWAT, inguinal white adipose tissue; eWAT, epididymal white adipose tissue; BAT, brown adipose tissue; GEL(au)FMT, gene expression level (arbitrary unit) of FMT; GEL(au)GF, gene expression level (arbitrary unit) of GF; BW(g)FMT, absolute body weight (gram) of FMT; BW(g)GF, absolute body weight (gram) of GF; BW(s)FMT, increasing ratio of body weight (slope) of FMT; and BW(s)GF, increasing ratio of body weight (slope) of GF.

### Gut microbiota that affects host gene expression

**Figure 5:**
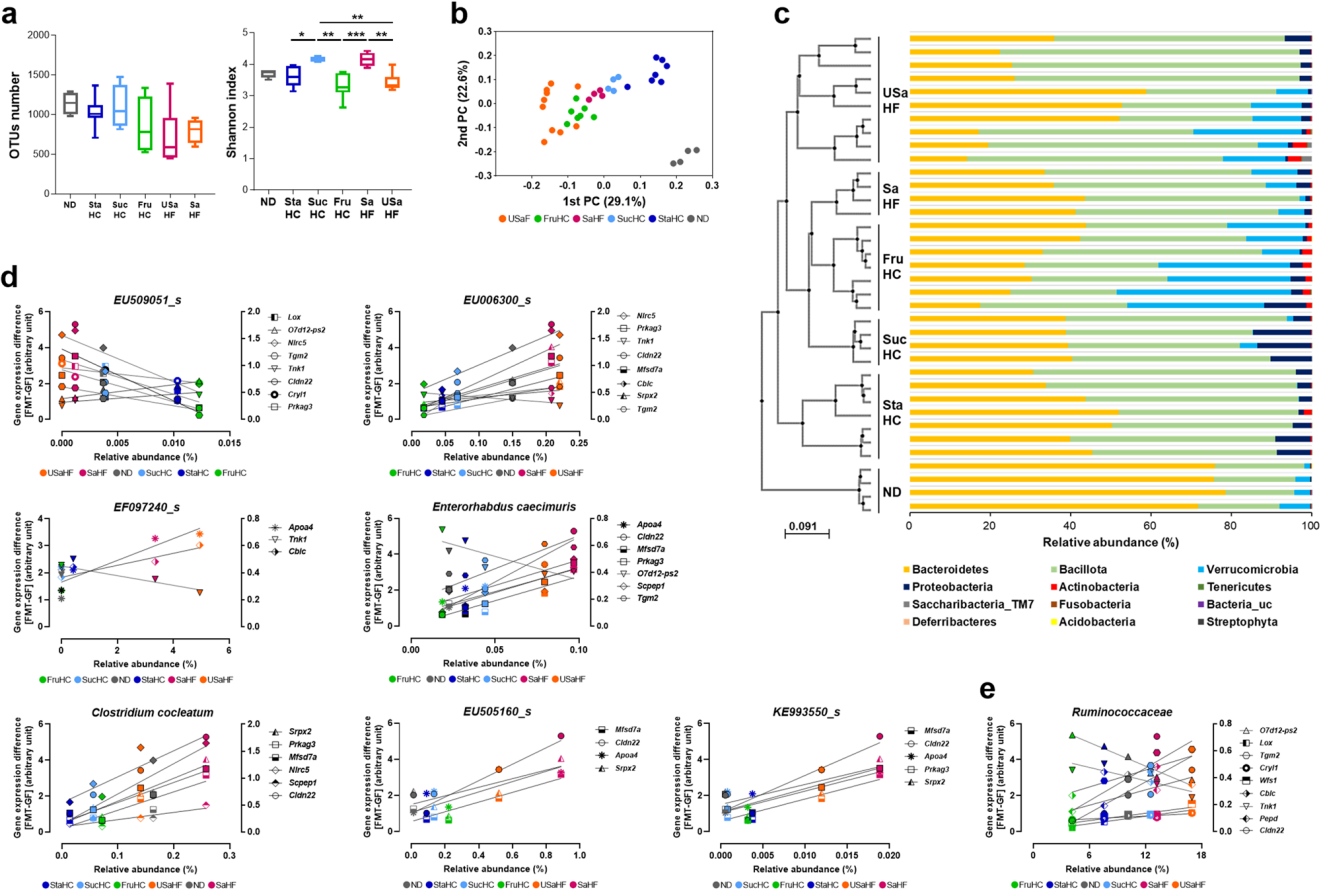
Microbiota analysis of fecal microbiota-transplant mice fed six different diets. (**a**) The box plots of alpha-diversity: left, OTUs number; right, Shannon index. The line shows the median, and the whiskers show the minimum and maximum. Asterisks (*) indicate significant differences (*p < 0.05, **p < 0.01, ***p < 0.001, two-way analysis of variance, followed by Tukey’s test). (**b**) Principal coordinate analysis plot based on species-level relative abundances using the Jensen–Shannon distance. (**c**) Taxonomic analysis: left, UPGMA clustering tree based on unweighted UniFrac distance; right, the relative phylum-level abundance map. N: ND = 4; StaHC = 7; SucHC = 4; FruHC = 7; SaHF = 4; USaHF = 10. (**d**) The correlation between the abundance of species-level gut microbiota and gene expression level (GEL) varied with diet. Lines were drawn using simple linear regression analysis. The x-axes indicate the average value of relative abundance. N: ND = 4; StaHC = 7; SucHC = 4; FruHC = 7; SaHF = 4; USaHF = 10. The left and right y-axes indicate the average value of subtracting the GEL of GF mice from that of FMT mice. N: ND = 4; StaHC = 7; SucHC = 4; FruHC = 7; SaHF = 4; USaHF = 4. *ND* normal diet, *StaHC*, high-starch diet, *SucHC* high-sucrose diet, *FruHC* high-fructose diet, *SaHF* saturated fatty acid-rich high-fat diet, *USaHF* unsaturated fatty acid-rich high-fat diet.

To identify the gut microbiota affecting the 17 host genes associated with weight regulation, we analyzed fecal 16S ribosomal RNA gene sequences of FMT mice for each of the six diets. The analysis of alpha-diversity based on the number of operational taxonomic units (OTUs) revealed that diet did not affect the number of taxa present (Fig. 5a, left); however, the Shannon diversity index was considerably affected by diet: microbiota in the SaHF and SucHC diet groups showed higher diversity than that in the USaHF and FruHC diet groups (Fig. 5a, right). Principal coordinate analysis based on Jensen–Shannon divergence showed that the ND diet group had a distinct microbial composition clustered separately from that of HF and HC diet groups (Fig. 5b). Figure 5c showed taxon-based analysis at the phylum level and hierarchical unweighted pair group method with arithmetic mean (UPGMA) clustering analysis at the species level. The ND diet group was characterized by an increased abundance of Bacteroidetes and a decreased abundance of Bacillota compared to other diet groups (*p* < 0.0001 and *p* < 0.01, respectively, Supplementary Table S5). The SucHC group was characterized by an increased abundance of Proteobacteria (*p* < 0.05). The FruHC group showed increased Verrucomicrobia (*p* < 0.001) and Actinobacteria (*p* < 0.01), and the USaHF group showed increased Bacillota (*p* < 0.05). From these gut microbiota, including 12 members at the phylum level, 29 at the class level, 68 at the order level, 126 at the family level, 336 at the genus level, and 1043 at the species level, we attempted to identify those associated with BW regulation, specifically those with changes in the expression of the 17 host genes mentioned earlier. Briefly, we hypothesized that the higher the abundance of gut microbiota, the greater the difference in host gene expression between FMT and GF mice (described as [GEL (au) FMT − GEL (au) GF] in Table 2, calculated as gene expression-arbitrary units in Supplementary Fig. S3). The same is true in the opposite direction as well. As both the gut microbiota abundance and GEL varied with diet, we evaluated the correlation coefficient between the two in the same diet group. Figure 5d shows the species-level gut microbiota with absolute value of correlation coefficients > 0.8. *EU509051_s* and *EU006300_s* correlated with eight of the 17 host genes associated with gut microorganism-mediated BW regulation (Table 2, bold letter), while *Enterohabdus caecimuris* correlated with seven genes, *Clostridium cocleatum* with six genes, *KE993550_s* with five genes, *EU505160_s* with four genes, and *EF097240_s* with three genes. Besides the species-level gut microorganisms, the family-level member *Ruminococcaceae* was correlated with nine genes (Fig. 5e). Furthermore, 35% (28 of 81 species) of the species-level microorganisms with an absolute value of correlation coefficient > 0.7 belonged to *Ruminococcaceae*.

### Gut microbiota affecting BW

**Figure 6:**
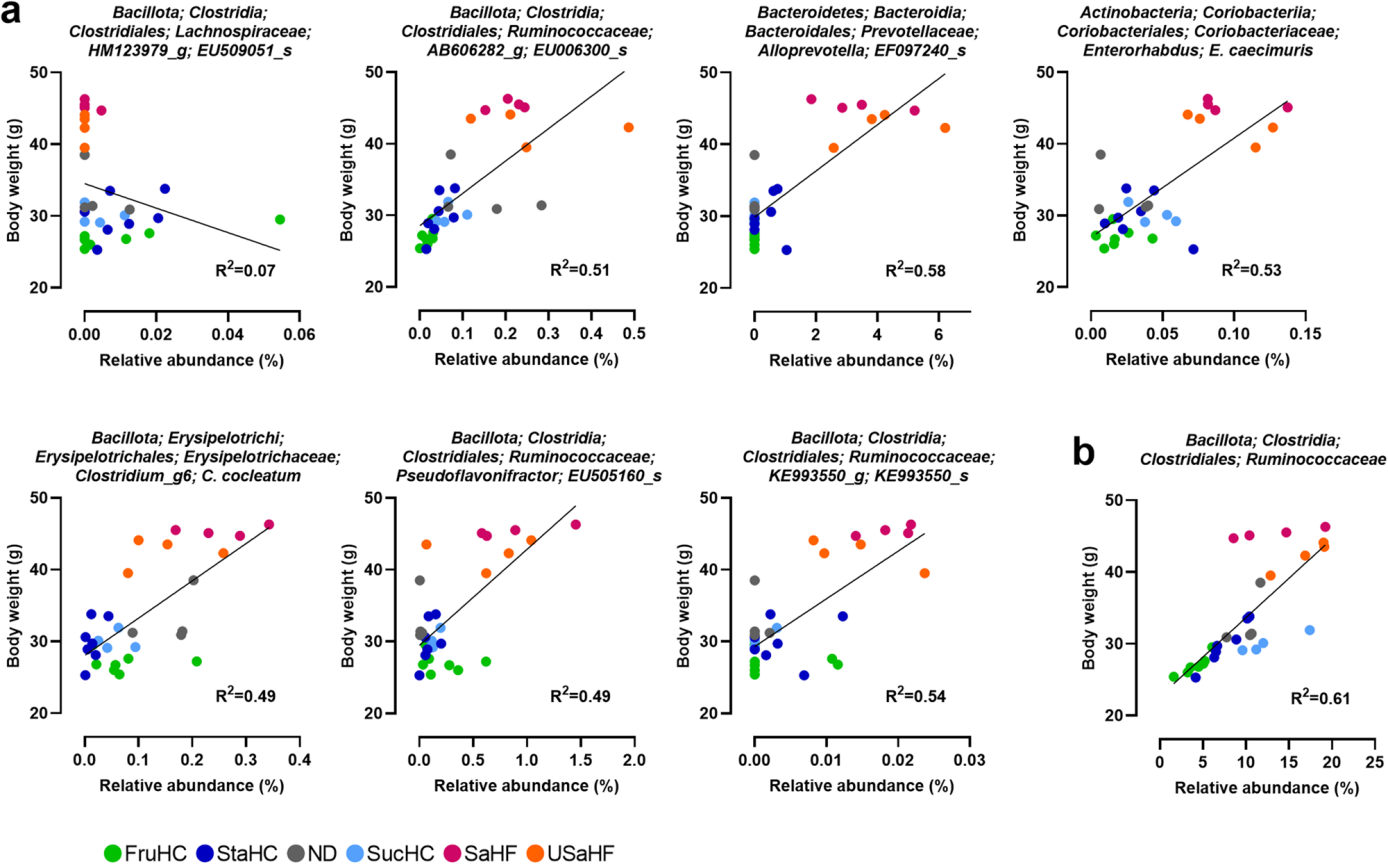
Body weight-associated microbiota. Correlation between the body weight and abundance of (**a**) microbiota species and (**b**) *Ruminococcaceae*. The lines were drawn via simple linear regression analysis. The colors of the dots indicate mice fed the following diets: gray, normal diet (ND); dark blue, high-starch diet (StaHC); light blue, high-sucrose diet (SucHC); green, high-fructose diet (FruHC); red, saturated fatty acid-rich high-fat diet (SaHF); and orange, unsaturated fatty acid-rich high-fat diet (USaHF).

We determined whether the identified gut bacteria (Fig. 5d,e) were responsible for changes in BW, generating a correlation plot between microbiota abundance and absolute BW after an 8-week dietary period for each individual mouse (Fig. 6a). *EU509051_s* showed no correlation (R^2^ = 0.07) with BW, while *EU006300_s* (R^2^ = 0.51), *EF097240_s* (R^2^ = 0.58), *E. caecimuris* (R^2^ = 0.53), *C. cocleatum* (R^2^ = 0.49), *EU505160_s* (R^2^ = 0.49), and *KE993550_s* (R^2^ = 0.54) showed a positive correlation. Furthermore, the abundance of *Ruminococcaceae* (R^2^ = 0.61, Fig. 6b) had a stronger correlation with BW than that of any single species belonging to *Ruminococcaceae*, such as *EU006300_s*, *EU505160_s*, and *KE993550_s*.

## Discussion

The contribution of microbiota to host fat accumulation and consequent weight gain has been demonstrated in several studies using mice models and microbiota transplantation^20–23^, supported by the results of the present study as well. FMT mice gained more weight than GF mice (Fig. 1 and Supplementary Fig. S1). The subcutaneous adipose tissue weight (Fig. 2a), blood glucose (Fig. 4a), insulin (Fig. 4d), and leptin (Fig. 4h) levels, along with the fat droplet size in subcutaneous adipose tissue (Fig. 3a) and liver (Fig. 3b), were all greater in FMT mice than in GF mice. The extent to which the host was affected by the gut microorganisms varied with diet (Figs. 1, 2, 3, 4), and no difference in appetite or food preference was observed between FMT and GF mice (Fig. 1h), suggesting that the existence of gut microorganisms directly affects host metabolism.

Several functions of gut microbiota that affect host energy balance have been proposed^24^, including derivation of energy from food^14^ via fermentation and degradation of indigestible polysaccharides, increase in gene expression related to nutrient transport and storage, production of intestinal hormones that contribute to body fat accumulation^25,26^, and induction of metabolic abnormalities such as insulin resistance by endotoxin^27^. Data from our study suggest that the ability of gut microorganisms to break down food ingested by the host and provide surplus energy from the resulting metabolites may contribute to weight gain. The BWs of mice fed diets rich in monosaccharides, such as fructose, showed a minor difference depending on the presence or absence of gut microorganisms (Fig. 1d,g), while that of mice fed diets rich in disaccharides, such as sucrose, or polysaccharides, including starch, showed large differences (Fig. 1b,c,g). Major carbon sources that provide energy for bacteria growing in the human intestine include dietary starches, plant cell wall polysaccharides (fiber), and oligosaccharides^28^. Therefore, a diet containing substances preferentially metabolized by gut microorganisms, such as polysaccharides and disaccharides, will help them produce more metabolites that serve as energy sources for the host, leading to host weight gain. In contrast, ingestion of monosaccharides, which are less utilized by microorganisms, will provide less additional energy to the host, resulting in less impact on weight. Such an effect of the gut microbiota metabolism was also observed in a comparison of mice fed two different HF diets. The additional weight portion of mice fed an USaHF-rich diet was greater than that of those fed an SaHF-rich diet (Fig. 1e–g). Alcock and Lin^29^ reported that the composition of gut microbiota of mice fed saturated and unsaturated fatty acids differs considerably due to variations in the generated bacterial metabolites, resulting in different gene expression levels in the host and consequent physiological outcomes. Thus, as the function of gut microorganisms in host physiology depends on the type of energy source for both the microorganisms and host, the process of identifying obesity-associated microorganisms may require consideration of the nutritional substances that affect both.

We analyzed the effects of six diets on the BW (Fig. 1a–g) and gene expression (Tables 1 and 2 and Supplementary Fig. S3) of GF and FMT mice, along with their gut microbiota (Fig. 5a–c). DEG analysis in a total of seven tissues identified host genes affected by gut microorganisms under each diet, and DEGs that were common across all six diet groups were determined (Table 1). Based on the correlation coefficients between BW and the amount of gene expression fluctuation associated with gut microorganisms, seventeen DEGs were identified as host genes associated with gut microorganism-mediated weight regulation (Table 2). The identification of gut microbiota involved in BW regulation (Fig. 5d,e) was performed by evaluating the correlation coefficients between the amount of fluctuation in the expression of these 17 genes by the gut microorganisms and abundance of gut microbiota in the same diet group. Finally, we validated whether the identified gut microbiota contributed to body weight and found a positive correlation between the abundance of *EU006300_s*, *EF097240_s*, *E. caecimuris*, *KE993550_s*, *C. cocleatum*, and *EU505160 _s* and individual mouse BW (Fig. 6a). Notably, of all levels of microbiota analyzed (phylum, class, order, family, genus, and species), the family-level microbiota member *Ruminococcaceae* showed the highest positive correlation with BW, being more abundant in heavier mice and less so in lighter mice (Fig. 6b).

It is well known that a characteristic of the intestinal microbiota of individuals with obesity is a high proportion of the phylum Bacillota^30,31^. Moreover, the abundance of certain Bacillota, such as Mollicutes^32^ at the class level; *Lactobacillus*^33^ and *Staphylococcus*^34^ at the genus level; and *Faecalibacterium prausnitzii*^35^, *Ruminococcus bromii*^36^, *Lactobacillus reuteri*^37^, and *Staphylococcus aureus*^38^ at the species level has been reported to increase with obesity traits. Considering that *Ruminococcaceae* belongs to the phylum Bacillota (Bacillota; Clostridia; Clostridiales; *Ruminococcaceae*), its identification as a gut microbiota member involved in BW regulation in the present study is reasonable. Furthermore, species belonging to the family *Ruminococcaceae*, such as *EU006300_s*, *EU 505160_s*, and *KE993550_s*, were identified as gut bacteria involved in BW regulation; however, the association that was expressed as a correlation coefficient between bacterial abundance and BW was weaker than that for the *Ruminococcaceae* family with the sum of several species (R^2^ = 0.51, 0.49, 0.54 versus R^2^ = 0.61). Therefore, when developing an anti-obesity treatment targeting intestinal bacteria, it may be more effective to include several species belonging to the family *Ruminococcaceae* rather than targeting a single species.

The family *Ruminococcaceae* and the genus *Ruminococcus* are involved in alcohol metabolism^39^, adipokine metabolism^40^, cirrhosis^41^, acute-on-chronic liver failure^42^, allergy^43,44^, antibiotic biosynthesis^45^, inflammation^46^, and cardiovascular risk^47^. However, the involvement of these bacteria in BW changes is controversial, with reports stating that individuals with obesity have either an increased^48–50^ or decreased^51–53^ abundance of *Ruminococcaceae* or that it varies with species^54^. As for the mechanism by which *Ruminococcaceae* members are involved in host BW regulation, it has been suggested that they degrade various polysaccharides to produce short-chain fatty acids (SCFAs)^55^. Supplementation of dietary SCFAs has been reported to inhibit BW gain associated with changes in the expression of G-protein coupled receptor 43 (GPR43) and GPR41^56^. Nonetheless, the direct causal relationship among *Ruminoccaceae*, BW, and SCFAs is yet unclear. To explore the reason why *Ruminococcaceae* are considered BW-associated bacteria, we focused our discussion on *Cldn22*, which had the highest frequency of occurrence as a gene, showing a correlation coefficient > 0.8 (Fig. 5d) between the gut microbiota abundance and GEL of the host genes associated with gut microorganism-mediated BW regulation (Table 2).

Although the function of CLDN22 has not yet been elucidated, it is inferred to represent a component of the tight junction chain^57–59^ as it encodes a member of the claudin family^60,61^. Tight junctions are thought to function as physical and chemical barriers that prevent food components and intestinal bacteria from freely passing through intercellular spaces between the epithelial and endothelial cell sheets^62,63^. For example, the loss of intestinal *Cldn7* expression results in epithelial cell sloughing and spontaneous inflammation^64^; *Cldn2*-deficient mice have defective water and sodium reabsorption function at the tight junctions of kidney proximal tubules^65^, and decreased *Cldn1* expression in the skin disrupts skin barrier function^66^. As claudin, a tight junction protein, can function as a regulator of paracellular barrier permeability in organs such as the intestine^67^, we speculate that in visceral adipose tissue, eWAT, it exchanges certain nutrients with the adherent intestinal tissue using its barrier function. Our hypothesis regarding the mechanism of involvement of *Ruminococcaceae* and *Cldn22* in obesity is as follows. Dietary fat components downregulate *Cldn22*, resulting in the disruption of the barrier function, and nutrients stored in adipose tissue leak into the interstitial space, getting absorbed in the intestinal tract. If *Ruminococcaceae* members are present, the bacteria produce metabolites from these absorbed nutrients and they are transported to the liver and other tissues of the host body, generating additional energy, which in turn causes weight gain. The metabolites transported to eWAT further downregulate *Cldn22*, resulting in a cycle of additional disruption of the barrier function, leakage of nutrients, digestion and absorption of those nutrients in the intestine, as well as consequent weight gain. Our results showed that *Cldn22* expression in eWAT was decreased when the host consumed HF diet, regardless of the presence or absence of gut microorganisms. In addition, the extent of decreased expression was greater in FMT mice than in GF mice (Supplementary Fig. S3b). These results may be consistent with the finding that the extent of BW gain in FMT mice, in which the barrier function is presumed to be repeatedly disrupted, was greater than that in GF mice, in which barrier disruption presumably did not occur.

Will improving tight junction and barrier function by manipulating the proportion of *Ruminococcaceae* lead to weight loss and weight gain inhibition? The answer is: it probably depends on diet. *Cldn22* expression is decreased when the host consumes an HF diet, even in the absence of gut microorganisms. The HF diet-induced downregulation of *Cldn22* expression is considerably greater than the decrease caused by gut microorganisms in a low-fat diet (Supplementary Fig. S3b). Thus, as long as the host consumes an HF diet, manipulating the proportion of *Ruminococcaceae* to suppress the downregulation of *Cldn22* will not overcome the downregulation caused by fat intake; therefore, it will not inhibit weight gain. However, if the host diet mainly includes carbohydrates such as sucrose and starch and is low in fat, the decrease in *Cldn22* expression caused by diet does not occur in the first place. Hence, if the downregulation of *Cldn22* due to gut microorganisms is prevented by manipulating the proportion of *Ruminococcaceae*, it may be possible to inhibit weight gain. Accordingly, the extent to which the host dietary components alter both the abundance of intestinal microorganisms and host gene expression should be considered to appropriately judge their effect on host physiology. In this way, obesity-associated intestinal microorganisms can be identified and used for developing obesity prevention and intervention strategies.

In this study, we focused on the correlation between *Ruminococcaceae* and *Cldn22* to investigate gut microorganisms associated with obesity. However, we could not identify a direct causal relationship between *Ruminococcaceae* and *Cldn22.* This highlights the need for further studies to verify this relationship using *Cldn22* transgenic mice. Furthermore, this study has some limitations regarding the analysis of microbiota. The advantage of the EzBioCloud 16S rRNA database used in this study is its high accuracy in identifying 16S rRNA sequences at the species level. However, a limitation of this database is that it lacks sequence differences in some closely related species. Thus, other databases, including SILVA and Greengenes^68^, are potentially better alternatives that can provide a more comprehensive set of 16S rRNA genes. There are some discussions pertaining to the representation of microbial diversity. For instance, it is argued that Chao1 is useful in obtaining richness but is inferior in terms of beta-diversity^69^, that the Shannon and Simpson indices cannot be employed as measures of diversity on their own^70^, and that weighted UniFrac is better than unweighted UniFrac as it places more emphasis on the deeper parts of the phylogeny^71^. Recently, a novel pipeline for processing 16S rRNA amplicon datasets in diversity analyses has been introduced^72^. A variety of analysis methods will need to be utilized for the exploration of target gut microorganisms in the future.

## Methods

### Animals and experimental design

All experimental procedures were approved and performed in accordance with the Institutional Animal Care and Use Committee of the RIKEN Yokohama Campus and in compliance with the ARRIVE guidelines. Male GF mice (C57BL/6[Gf]; age, 7–9 weeks) were purchased from CLEA Japan Inc. (Shizuoka, Japan) and maintained in GF conditions in vinyl isolators with an alternating 12-h light/dark cycle at 23 °C with free access to food and water. After the acclimatization period, the mice were divided into two groups with similar mean body weights and standard deviations. Mice in one group were colonized via oral gavage with feces from specific pathogen-free C57BL/6N mice fed a standard chow diet (CLEA Rodent Diet CE-2: 12% of calories from fat, 59.1% of calories from carbohydrates, and 28.8% of calories from protein; CLEA Japan Inc.) or a high-fat diet (CLEA High Fat Diet 32 HFD32: 56.7% of calories from fat, 23.1% of calories from carbohydrates, and 20.0% of calories from protein; CLEA Japan). These were referred to as FMT mice, and the other group that was administered autoclaved feces was referred to as GF mice. Feces were suspended in phosphate-buffered saline (10% w/v) to create fecal slurries and orally gavaged using 10 mL/kg (250 μL maximum) of the prepared slurry. One day after oral administration, fecal samples from each group were Gram-stained to confirm bacterial colonization or sterility. Each group was fed gamma-ray-sterilized ND (CMF; Oriental Yeast Company, Ltd., Shiga, Japan), StaHC (D12450K; Research Diets Inc., New Brunswick, NJ, USA), SucHC (D07042201; Research Diets Inc.), FruHC (D08040107; Research Diets Inc.), USaHF (HFD32; CLEA Japan Inc.), and SaHF (D12492; Research Diets Inc.) diets and then kept in isolators. The basic compositions of the six different diets and fatty acid compositions of the HF diets are shown in Supplementary Tables S1 and S2, respectively. After 8 or 10 weeks of feeding, the mice were euthanized under isoflurane anesthesia, blood was collected, and organs including the iWAT, eWAT, BAT, muscle, liver, duodenum, and ileum were quickly removed, weighed, and submerged in RNAlater solution (Thermo Fisher Scientific, Waltham, MA) at 4 °C for 20 h and stored at − 20 °C. Fecal samples were individually collected the day before dissection and stored at − 80 °C until use.

### Plasma parameters

Blood glucose level was determined using a compact glucose analyzer (Glutest Sensor, Sanwa Kagaku, Nagoya, Japan). Plasma insulin (Morinaga Institute of Biological Science, Kanagawa, Japan), leptin (R&D Systems, Minneapolis, MN, USA), and adiponectin (Otsuka Pharmaceutical Co., Ltd., Tokyo, Japan) levels were measured using enzyme-linked immunosorbent assay kits. Plasma TG, T-Cho, HDL, NEF, ALT, and AST levels were measured using reagents from Wako Pure Chemical Industries, Ltd. (Osaka, Japan). All assays were performed according to the manufacturer’s instructions.

### RNA sequencing

#### Tissue preparation and isolation of RNA

Minced tissues were homogenized in Sepasol RNAI solution (Nacalai Tesque, Kyoto, Japan) using a TissueLyser LT instrument (Qiagen, Hilden, Germany) set at 50 strokes/s for 5 min. The homogenate of adipose tissues was centrifuged at 3000×*g* for 10 min, and the bottom layer was transferred to a new tube to separate the fat from the upper layer. Chloroform was then added, and the vortexed sample was centrifuged at 14,000×*g* for 10 min to separate the RNA phase. The RNA phase was transferred to a new tube and subjected to total RNA purification using QIAcube and the RNeasy kit (Qiagen); quality analysis was performed by TapeStation (Agilent Technologies, Santa Clara, CA, USA) using RNA ScreenTape (Agilent).

#### Library construction and sequencing

Libraries were generated using the NEBNext Ultra RNA Library Prep Kit for Illumina (New England Biolabs, Ipswich, MA, USA). The poly-A labeled fraction of total RNA (250 ng) was enriched using magnetic poly-T beads. First- and second-strand cDNAs were synthesized using random hexamer primers (included in the kit), M-MuLV reverse transcriptase, DNA polymerase I, and RNase H, followed by the conversion of overhangs to blunt the ends. DNA fragments were ligated with NEBNext adaptors and size-fractionated using the AMPure XP system (Beckman Coulter, Inc., Brea, CA, USA) before treatment with USER enzyme (New England Biolabs). Polymerase chain reaction (PCR) amplification was performed with universal and index primers using Phusion high-fidelity DNA polymerase. The PCR products were purified using the AMPure XP system, and the quality of the library was assessed using the TapeStation system (Agilent). Pooled libraries were sequenced on an Illumina HiSeq 2500 platform to obtain 50 bp single-end reads.

#### Read mapping and quantification of gene expression level

Reads were mapped to genes in the reference mouse genome (UCSC mm10) and assembled into transcripts, whose abundance was estimated as the expected number of fragments per kilobase per millions of base pairs sequenced (FPKM) using Cufflinks (v 1.3.0)^73^. Bowtie (v 0.12.7)^74^ was used to build an index of the reference genome, and TopHat (v 1.4.0)^75^ was used to align reads.

#### DEG analysis

Data were analyzed using Strand NGS (v 2.7, Strand Life Sciences, Bangalore, India). DESeq was used to compare pairs of sample groups that included at least four biological replicates for each group. All genes were tested to obtain the corresponding *p*-values, followed by multiple testing corrections using the Benjamini and Hochberg method to acquire the corrected *p*-value (*q*). Comparing the significance of differences in GELs between groups was analyzed using the Tukey’s post-hoc test on normalized FPKM.

### Microbiota analysis

#### DNA isolation and sequencing

Fecal samples were individually collected and stored at − 80 °C until DNA extraction was performed using the QIAamp PowerFecal DNA Isolation kit (Qiagen) with a bead-beating step (5 mm stainless steel beads, Qiagen) according to the manufacturer's instructions. PCR amplification was performed using the following set of primers: 341F (ִִ5′-TCGTCGGCAGCGTCAGATGTGTATAAGAGACAG-CCTACGGGNGGCWGCAG-3′) and 805R (5′-GTCTCGTGGGCTCGG-AGATGTGTATAAGAGACAGGACTACHVGGGTATCTAATCC-3′) to target the V3–V4 regions of the 16S rRNA gene, and the amplified products were purified using a QIAquick PCR purification kit (Qiagen) according to the manufacturer's instructions. Equal concentrations of purified products were pooled together, and short fragments (non-target products) were removed using an Ampure beads kit (Agencourt Bioscience, Beverly, MA, USA). The quality and product size were assessed on a 2100 Bioanalyzer (Agilent). Mixed amplicons were pooled, and sequencing was performed by ChunLab Inc. (Seoul, South Korea) using the Illumina MiSeq system (Illumina, San Diego, CA, USA).

#### MiSeq pipeline

The raw reads were submitted to a quality check, and low-quality (< Q25) reads were filtered out using Trimmomatic 0.32. The paired-end sequences (250 bp) were then merged using PANDAseq^76^. Primers were trimmed using a Chunlab in-house program (Chunlab, Inc.) at a similarity cut-off of 0.8; the sequences were denoised using the DUDE-Seq to correct the sequencing errors. From all quality-controlled sequences, 20,000 reads were randomly selected, and UCHIME and the 16S database in the EzBioCloud^77^ were used to identify chimera reads with a best-hit similarity rate below 97%. Taxonomic assignment was performed based on the EzBioCloud database^78^ (http://ezbiocloud.net), and sequence similarity was calculated via pairwise alignment. Sequences that matched the reference sequence by > 97% similarity in EzBioCloud were considered “identified” at the species level. The sequences that did not match with sequences in the EzBioCloud 16S database were then clustered using cluster database at high identity with tolerance (CD-HIT) and UCLUST tools with a 97% similarity boundary. The species identified via the EzBioCloud 16S database and OTUs obtained using CD-HIT and UCLUST tools were combined to form the final set of OTUs, and the remaining singletons were excluded. Other sequence similarity cut-offs were genus (97% > x ≥ 94.5%), family (94.5% > x ≥ 86.5%), order (86.5% > x ≥ 82%), class (82% > x ≥ 78.5%), and phylum (78.5% > x ≥ 75%).

#### Data analysis

16S rRNA reads were analyzed using EzBioCloud. Differences in the alpha-diversity, including the number of OTUs, richness (Chao1), and diversity (Shannon index), were investigated in the six diet groups. Differences between groups were tested using the ANOVA and post-hoc Tukey's tests. The beta diversity was visualized using Jensen–Shannon divergence-based principal coordinate analysis and UPGMA phylogenetic tree based on unweighted UniFrac distance. The taxonomic composition of the fecal microbiota was investigated at the levels of phylum, class, order, genus, and species.

### Histological analysis

Samples were fixed for 20 h in Mildform 10N (Wako), embedded in paraffin, and then subjected to hematoxylin and eosin staining (Meyer’s hematoxylin solution and 1% eosin, Muto Pure Chemicals Co., Ltd., Tokyo, Japan) as well as immunohistochemistry using primary antibodies against F4/80 (Cedarlane Laboratories, Ontario, Canada). Oil Red O staining (Nacalai Tesque) was performed on frozen sections. For the image analysis, a microscope BZ-X710 (Keyence, Osaka, Japan), BZ-X Viewer version 1.3.1.1 (Keyence), and BZ-X Analyzer version 1.3.1.1 (Keyence) were used.

### Statistical analysis

Statistical analyses were carried out using GraphPad Prism 8 software (GraphPad Software, San Diego, CA, USA). Quantitative two-group data were analyzed using an unpaired two-tailed *t* test. A comparison of data with two factors was performed using a two-way ANOVA and a one-way ANOVA, followed by Tukey’s test.

## Supplementary Information

Supplementary Information.

## Data Availability

The sequencing data obtained in this study were deposited in the DDBJ Sequence Read Archive (DRA) under accession numbers DRA014969, DRA015107, DRA015108, DRA015170, DRA015171, DRA015172, DRA015173 (RNA-seq), and DRA015109 (16S rRNA). The data can be found in the webpage: https://ddbj.nig.ac.jp/.
