# Supplementary material for: Exploring body weight-influencing gut microbiota by elucidating the association with diet and host gene expression
Source: Sci Rep. 2023 Apr 5;13:5593. doi: 10.1038/s41598-023-32411-z (PMC10076326; doi:10.1038/s41598-023-32411-z)
Supplement: Supplementary file 1 — Supplementary Information. [file 41598_2023_32411_MOESM1_ESM.pdf]

Exploring Body Weight-Influencing Gut Microbiota by Elucidating the Association with Diet and Host Gene Expression

Shino Nemoto, Tetsuya Kubota & Hiroshi Ohno

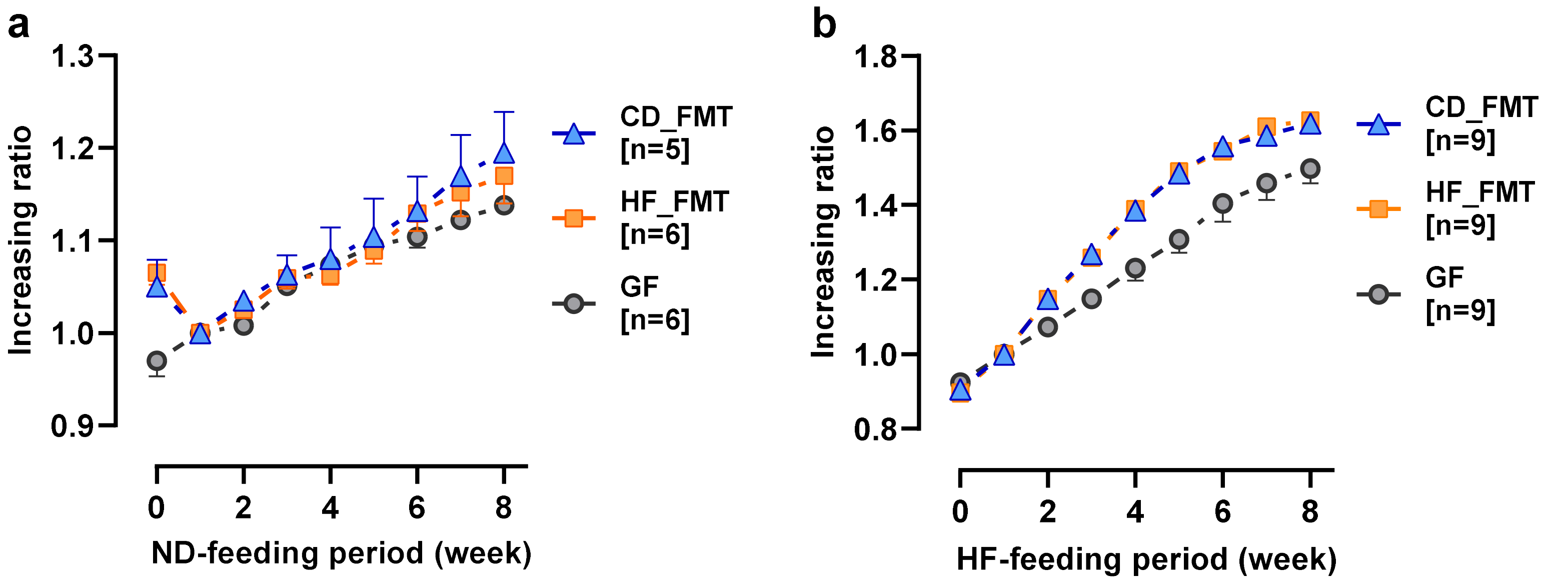

Supplementary Figure S1. Difference in increasing ratio of body weight between germ-free (GF) and fecal microbiota-administered (FMT) mice. Mice were fed (a) a normal diet (ND) (n=5 or 6) or (b) a high-fat diet (HF) (n=9) for eight weeks. CD_FMT (triangle), GF mice administered with feces from standard chow diet (CD)-fed mice; HF_FMT (square), GF mice administered feces from HF-fed mice; and GF (circle), GF mice administered autoclaved feces. The body weight at week 1 after administration of feces was set as "1". Values are means + or - SEM.

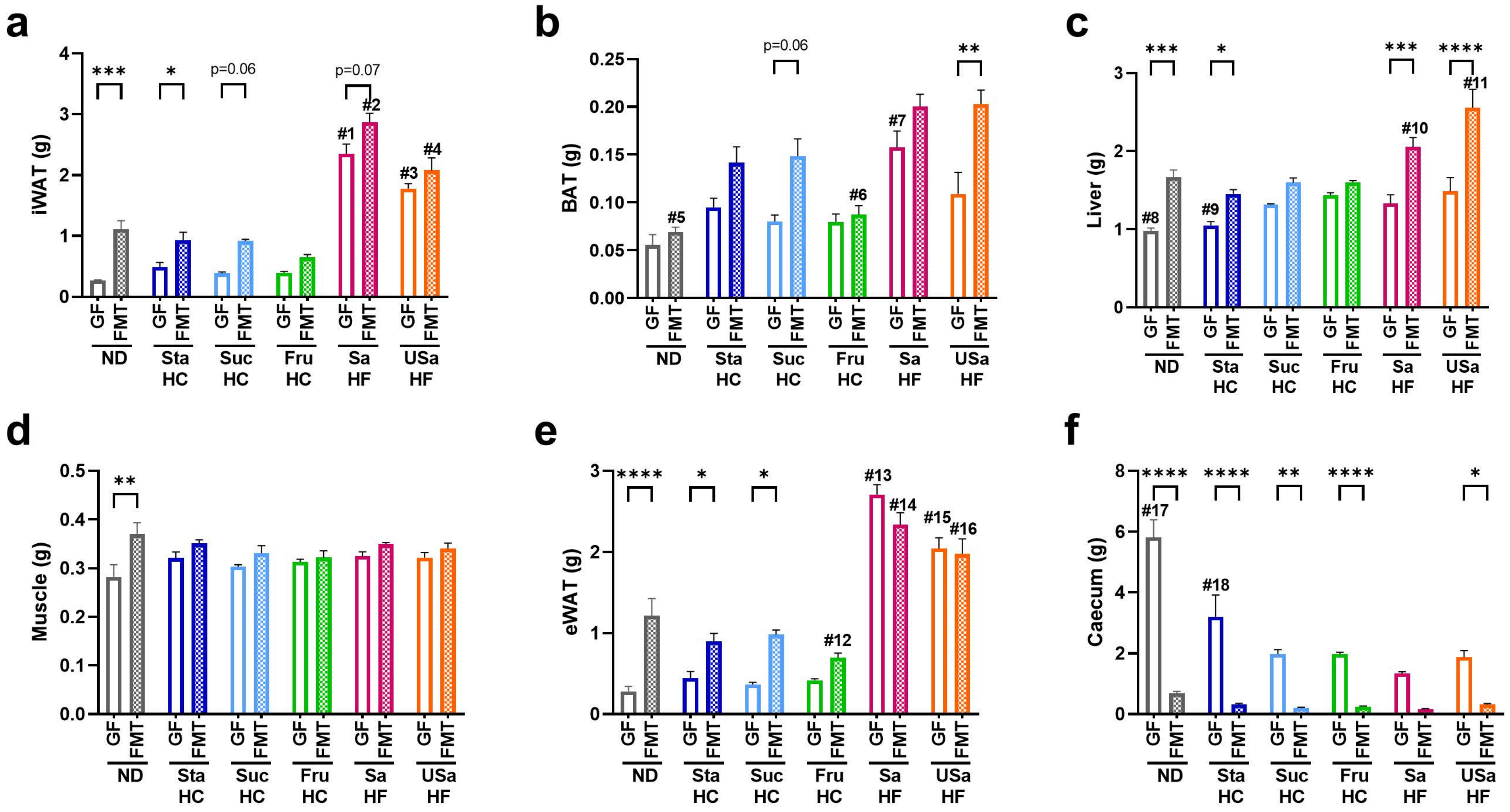

Supplementary Figure S2. Difference in tissue weights between germ-free mice (GF) and fecal microbiota-administered mice (FMT) fed six different diets. (a) inguinal white adipose tissue (iWAT), (b) brown adipose tissue (BAT), (c) liver (d) skeletal muscle (muscle), (e) epididymal white adipose tissue (eWAT), and (f) caecum with content. Values are absolute weight (gram) and are means + SEM (n=4 or 7). Multiple comparisons were analyzed using two-way ANOVA, followed by Tukey's test. Asterisks (*) indicate significant differences between GF and FMT (*$p < 0.05$, **$p < 0.01$, ***$p < 0.001$, and ****$p < 0.0001$). The # denotes the significances between the diet groups, and the statistical results for # numbers correspond to the # numbers marked in Supplementary Table 4. ND, normal diet; StaHC, high-starch diet; SucHC, high-sucrose diet; FruHC, high-fructose diet; SaHF, saturated fatty acids-rich high-fat diet; USaHF, unsaturated fatty acids-rich high-fat diet.

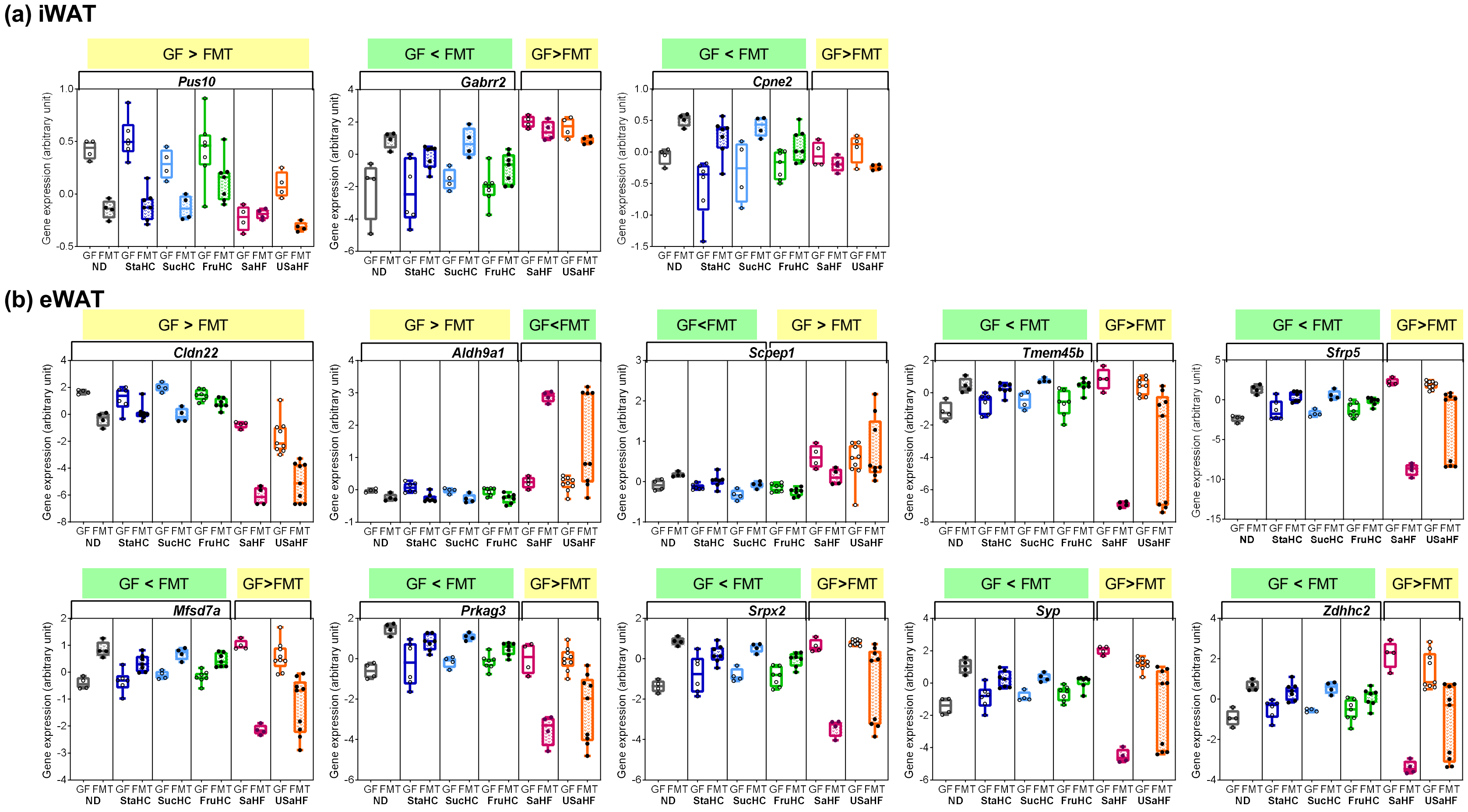

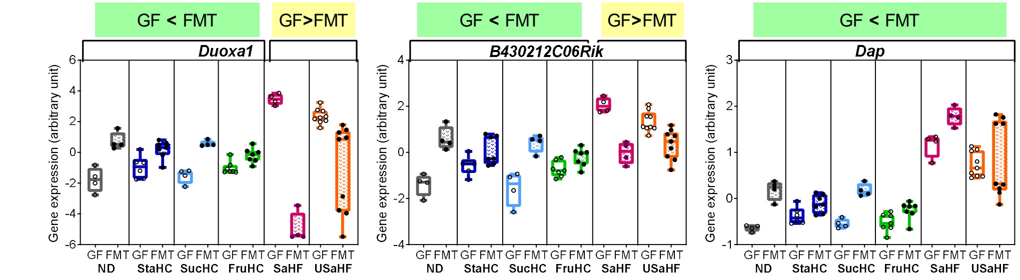

Supplementary Figure S3. Gene expression levels of differentially expressed genes between germ-free (GF) and fecal microbiota-administered (FMT) mice fed six different diets. (a) inguinal white adipose tissue (iWAT), (b) epididymal white adipose tissue (eWAT), (c) brown adipose tissue (BAT), (d) skeletal muscle (muscle), (e) liver, (f) duodenum, and (g) ileum. ND, normal diet; StaHC, high-starch diet; SucHC, high-sucrose diet; FruHC, high-fructose diet; SaHF, saturated fatty acids-rich high-fat diet; USaHF, unsaturated fatty acids-rich high-fat diet.

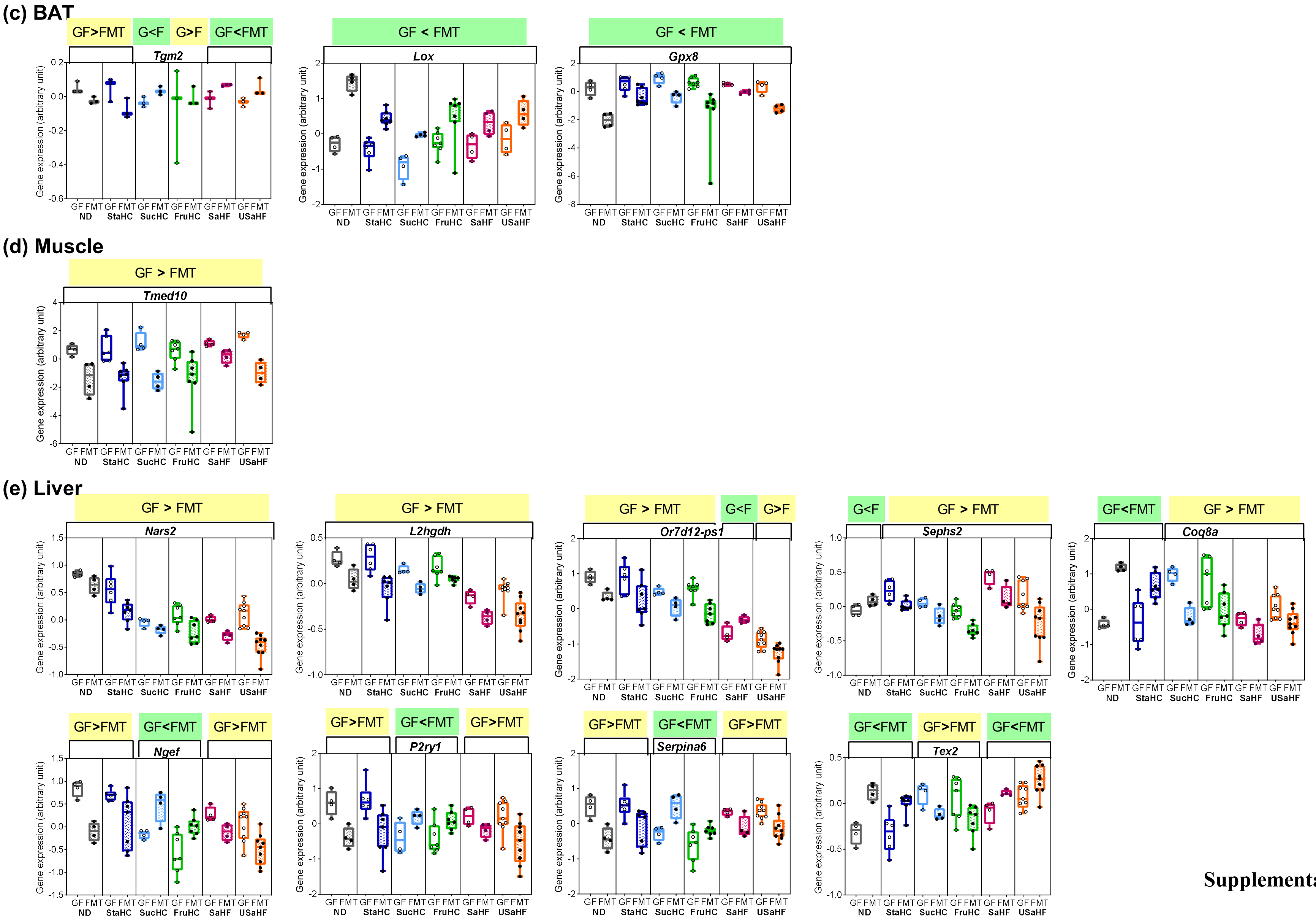

Supplementary Figure S3.

(e) Liver

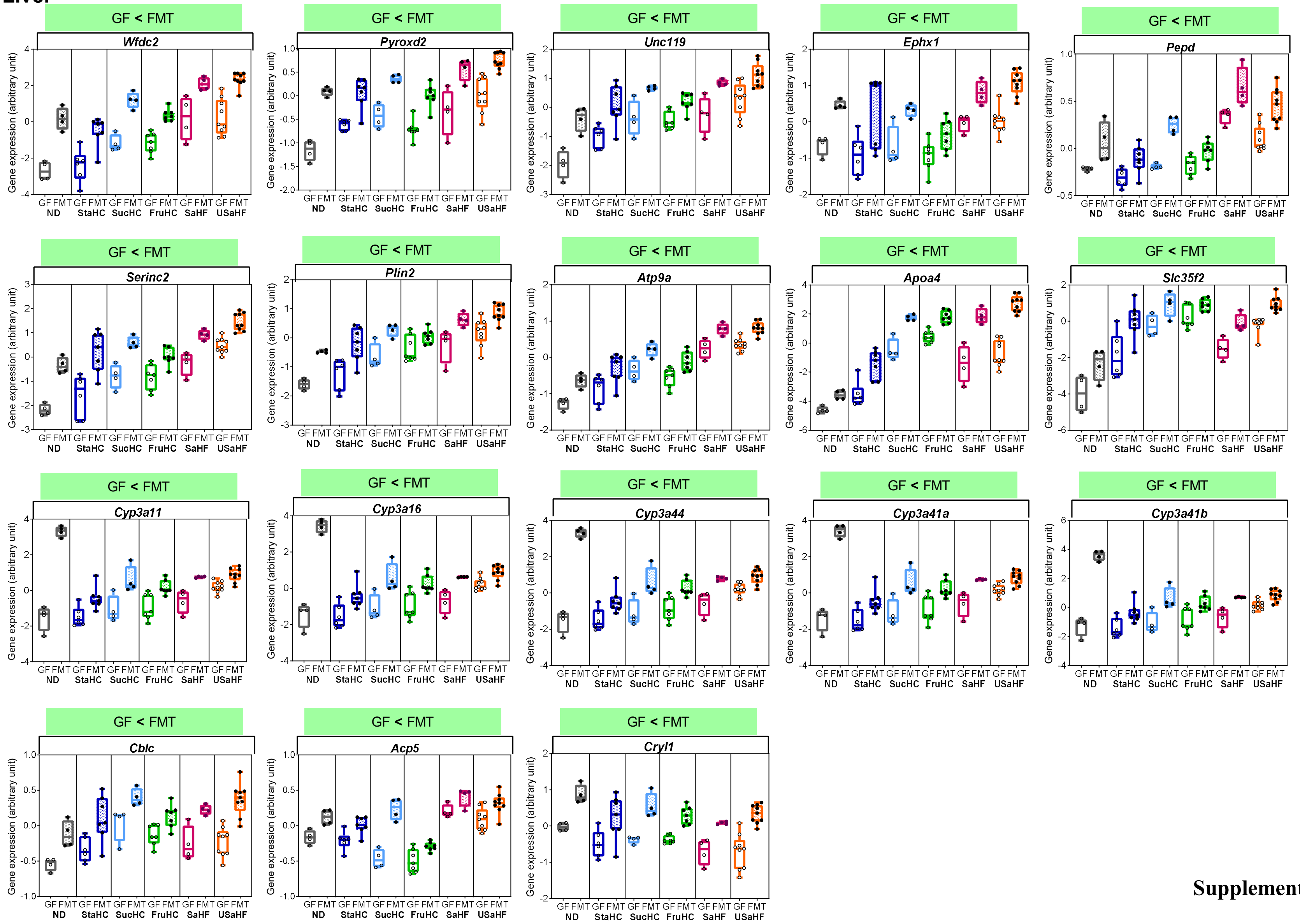

Supplementary Figure S3.

(f) Duodenum

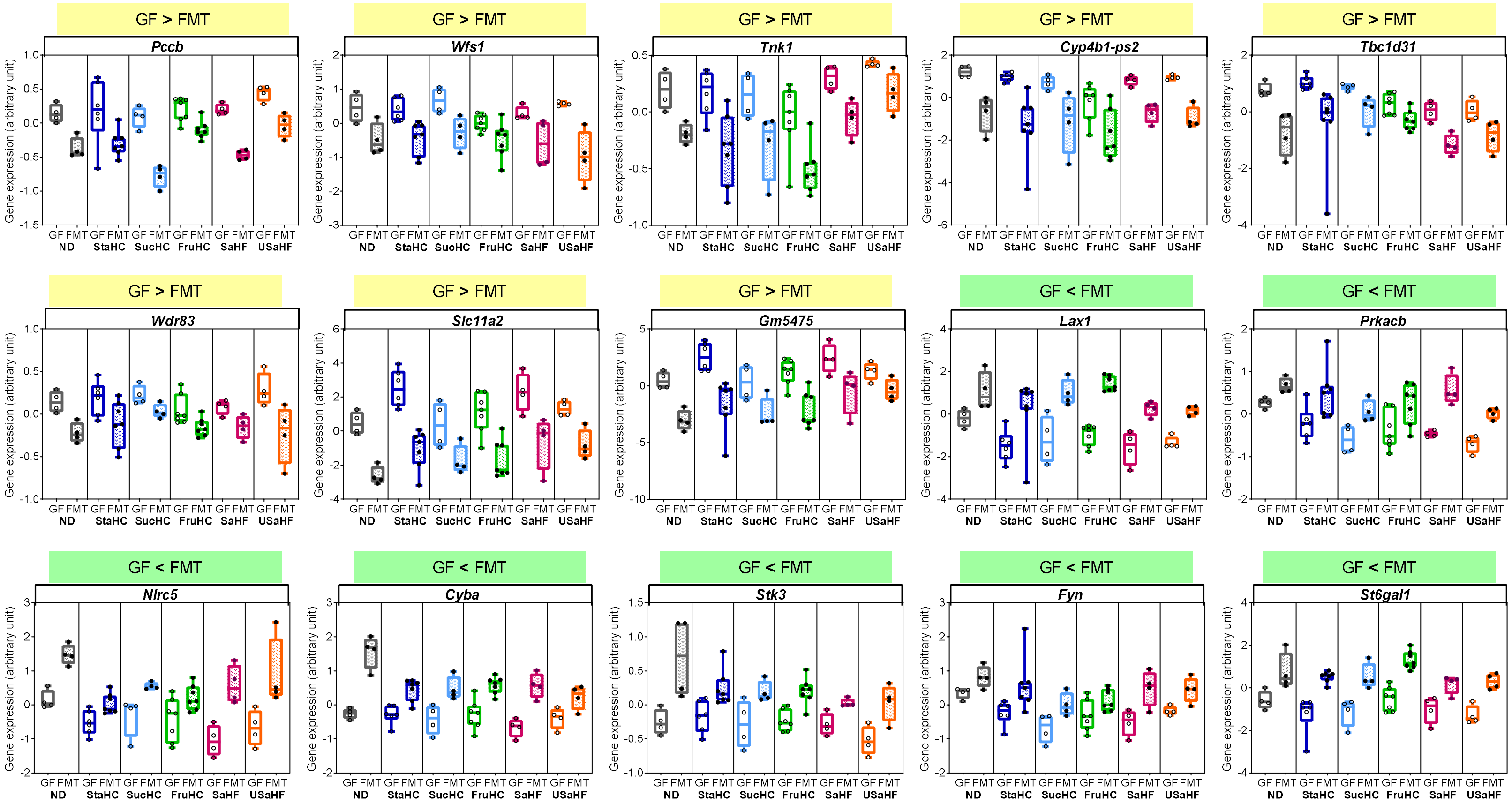

(g) Ileum

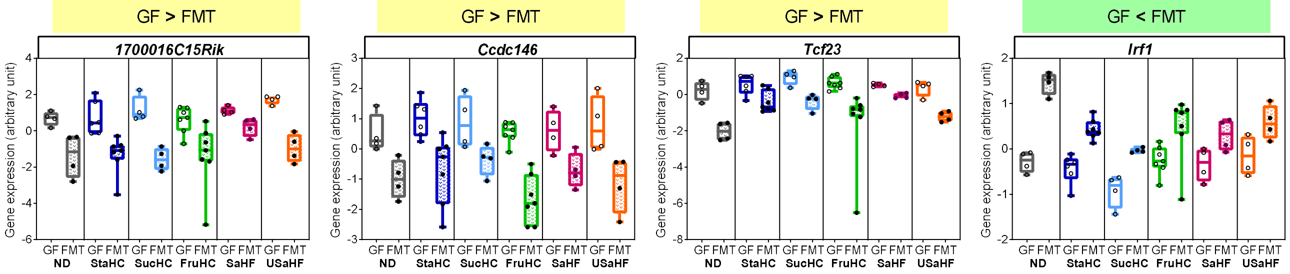

Supplementary Figure S3.

Supplementary Table S1. **Basic composition of six different diets**.

		Normal	High carbohydrate (HC)			High fat (HF)	
		(ND)	high-Starch (StaHC)	high-Sucrose (SucHC)	high-Fructose (FruHC)	Satura -ted fatty acid-rich (SaHF)	Unsatura-ted fatty acid-rich (USaHF)
Moisture	g /100g	8	1	1	1	8	7
NFE	g /100g	47	67	67	67	31	31
Fat	g /100g	8	4	4	4	33	31
Protein	g /100g	28	19	19	19	22	25
Fiber	g /100g	6	5	5	5	3	2
Ash	g /100g	3	4	4	4	4	4
Energy	kcal/100g	372	385	385	380	523	506
Carbohydrate	kcal%	50	70	70	70	20	23
Fat	kcal%	20	10	10	10	60	57
Protein	kcal%	30	20	20	20	20	20

Supplementary Table S2. Fatty acid compositions of high-fat diets (HF).

		High-fat diet	
		Saturated fatty acid-rich (SaHF)	Unsaturated fatty acid-rich (USaHF)
Saturated (%)		32	22
Monounsaturated (%)		36	67
Polyunsaturated (%)		32	10
Fatty acid		(%)	(%)
Capric	C10	0.04	0
Lauric	C12	0.08	0
Myristic	C14	1.1	1.1
Myristoleic	C14:1	0	0.31
Pentadecane	C15	0.08	0.09
Palmitic	C16	20	13
Palmitoleic	C16:1	1.3	1.2
Heptadecane	C17	0.35	0.41
Heptadecene	C17:1	0	0.31
Stearic	C18	11	7.5
Oleic	C18:1	34	64
Linoleic	C18:2	29	10
Linolenic	C18:3	2.0	0.19
Arachidic	C20	0.16	0.31
Icosenoic	C20:1	0.59	0.31
Icosadienoic	C20:2	0.79	0
Icosatrienoic	C20:3	0.12	0
Arachidonic	C20:4	0.28	0
Behenic	C22	0	0.19
Docosapentaenoic	C22:5	0.08	0
Unknown		0	0.82

SupplementaryTable S3. **Difference in tissue weight between the diet groups.**

Data indicate p-value analyzed by two-way analysis of variance and Tukey's test. Bold letter, $p < 0.05$.

#Numbers correspond to the #numbers marked in Figure 2.

iWAT			ND	StaHC	SucHC	FruHC	SaHF	USaHF
GF		ND		0.470	0.931	0.889	**<0.0001**	**<0.0001**
		StaHC	0.470		>0.99	>0.99	**<0.0001**	**<0.0001**
		SucHC	0.931	>0.99		>0.99	**<0.0001**	**<0.0001**
		FruHC	0.889	>0.99	>0.99		**<0.0001**	**<0.0001**
	#1	SaHF	**<0.0001**	**<0.0001**	**<0.0001**	**<0.0001**		0.420
	#3	USaHF	**<0.0001**	**<0.0001**	**<0.0001**	**<0.0001**	0.420	
FMT		ND		>0.99	>0.99	0.147	**<0.0001**	**0.006**
		StaHC	>0.99		>0.99	0.444	**<0.0001**	**<0.0001**
		SucHC	>0.99	>0.99		0.643	**<0.0001**	**0.004**
		FruHC	0.147	0.444	0.643		**<0.0001**	**<0.0001**
	#2	SaHF	**<0.0001**	**<0.0001**	**<0.0001**	**<0.0001**		**0.014**
	#4	USaHF	**0.006**	**<0.0001**	**0.004**	**<0.0001**	**0.014**	

Muscle			ND	StaHC	SucHC	FruHC	SaHF	USaHF
GF		ND		0.260	0.170	0.064	0.110	0.724
		StaHC	0.260		>0.99	>0.99	**<0.0001**	**0.0008**
		SucHC	0.170	>0.99		>0.99	**<0.0001**	**0.0008**
		FruHC	0.064	>0.99	>0.99		**<0.0001**	**<0.0001**
	#10	SaHF	0.110	**<0.0001**	**<0.0001**	**<0.0001**		0.990
	#12	USaHF	0.724	**0.0008**	**0.0008**	**<0.0001**	0.990	
FMT		ND		>0.99	>0.99	0.975	>0.99	**0.0005**
		StaHC	>0.99		0.956	>0.99	<0.0001	<0.0001
		SucHC	>0.99	0.956		0.853	0.0003	0.002
		FruHC	0.975	>0.99	0.853		<0.0001	<0.0001
	#11	SaHF	>0.99	**<0.0001**	**0.0003**	**<0.0001**		>0.99
	#13	USaHF	**0.0005**	**<0.0001**	**0.002**	**<0.0001**	>0.99	

BAT			ND	StaHC	SucHC	FruHC	SaHF	USaHF
GF		ND		0.319	0.760	0.747	0.132	0.969
		StaHC	0.319		>0.99	>0.99	>0.99	>0.99
		SucHC	0.760	>0.99		>0.99	>0.99	>0.99
		FruHC	0.747	>0.99	>0.99		0.930	>0.99
		SaHF	0.132	>0.99	>0.99	0.930		0.941
		USaHF	0.969	>0.99	>0.99	>0.99	0.941	
FMT	#5	ND		**0.0008**	**0.014**	0.638	**0.017**	**0.003**
		StaHC	**0.0008**		>0.99	0.770	>0.99	>0.99
		SucHC	**0.014**	>0.99		0.092	>0.99	>0.99
		FruHC	0.638	0.770	0.092		0.575	0.157
		SaHF	**0.017**	>0.99	>0.99	0.575		>0.99
		USaHF	**0.003**	>0.99	>0.99	0.157	>0.99	

eWAT			ND	StaHC	SucHC	FruHC	SaHF	USaHF
GF		ND		0.856	0.989	0.850	**<0.0001**	**<0.0001**
		StaHC	0.856		>0.99	>0.99	**<0.0001**	**<0.0001**
		SucHC	0.989	>0.99		>0.99	**<0.0001**	**<0.0001**
		FruHC	0.850	>0.99	>0.99		**<0.0001**	**<0.0001**
	#14	SaHF	**<0.0001**	**<0.0001**	**<0.0001**	**<0.0001**		0.206
	#16	USaHF	**<0.0001**	**<0.0001**	**<0.0001**	**<0.0001**	0.206	
FMT		ND		0.715	>0.99	0.111	**0.012**	0.232
		StaHC	0.715		>0.99	0.968	**<0.0001**	**0.0004**
		SucHC	>0.99	>0.99		0.670	**0.0007**	**0.024**
		FruHC	0.111	0.968	0.670		**<0.0001**	**<0.0001**
	#15	SaHF	**0.012**	**<0.0001**	**0.0007**	**<0.0001**		0.987
	#17	USaHF	0.232	**0.0004**	**0.024**	**<0.0001**	0.987	

Liver			ND	StaHC	SucHC	FruHC	SaHF	USaHF
GF		ND		0.973	**<0.0001**	**<0.0001**	>0.99	0.805
		StaHC	0.973		**0.001**	**<0.0001**	0.519	>0.99
	#6	SucHC	**<0.0001**	0.001		0.982	**<0.0001**	**0.004**
	#7	FruHC	**<0.0001**	**<0.0001**	0.982		**<0.0001**	**<0.0001**
		SaHF	>0.99	0.519	**<0.0001**	**<0.0001**		0.272
		USaHF	0.805	>0.99	**0.004**	**<0.0001**	0.272	
FMT		ND		>0.99	>0.99	**0.025**	0.714	**0.036**
		StaHC	>0.99		0.754	**0.0002**	0.964	**0.0007**
		SucHC	>0.99	0.754		0.284	0.175	0.292
	#8	FruHC	**0.025**	**0.0002**	0.284		**<0.0001**	>0.99
		SaHF	0.714	0.964	0.175	**<0.0001**		**<0.0001**
	#9	USaHF	**0.036**	**0.0007**	0.292	>0.99	**<0.0001**	

Caecum			ND	StaHC	SucHC	FruHC	SaHF	USaHF
GF	#18	ND		**<0.0001**	**<0.0001**	**<0.0001**	**<0.0001**	**<0.0001**
	#19	StaHC	**<0.0001**		0.198	**0.049**	**<0.0001**	**0.0009**
		SucHC	**<0.0001**	0.198		>0.99	0.110	0.766
		FruHC	**<0.0001**	**0.049**	>0.99		0.066	0.730
		SaHF	**<0.0001**	**<0.0001**	0.110	0.066		0.983
		USaHF	**<0.0001**	**0.0009**	0.766	0.730	0.983	
FMT		ND		>0.99	>0.99	>0.99	0.980	>0.99
		StaHC	>0.99		>0.99	>0.99	>0.99	>0.99
		SucHC	>0.99	>0.99		>0.99	>0.99	>0.99
		FruHC	>0.99	>0.99	>0.99		>0.99	>0.99
		SaHF	0.980	>0.99	>0.99	>0.99		>0.99
		USaHF	>0.99	>0.99	>0.99	>0.99	>0.99	

SupplementaryTable S4. **Difference in tissue weight between the diet groups.**

Data indicate p-value analyzed by two-way analysis of variance and Tukey's test. Bold letter, $p < 0.05$.

#Numbers correspond to the #numbers marked in Supplementary Figure S2.

iWAT			ND	StaHC	SucHC	FruHC	SaHF	USaHF
GF		ND		0.928	>0.99	>0.99	**<0.0001**	**<0.0001**
		StaHC	0.928		>0.99	>0.99	**<0.0001**	**<0.0001**
		SucHC	>0.99	>0.99		>0.99	**<0.0001**	**<0.0001**
		FruHC	>0.99	>0.99	>0.99		**<0.0001**	**<0.0001**
	#1	SaHF	**<0.0001**	**<0.0001**	**<0.0001**	**<0.0001**		**0.029**
	#3	USaHF	**<0.0001**	**<0.0001**	**<0.0001**	**<0.0001**	**0.029**	
FMT		ND		0.974	0.982	0.064	**<0.0001**	**<0.0001**
		StaHC	0.974		>0.99	0.438	**<0.0001**	**<0.0001**
		SucHC	0.982	>0.99		0.729	**<0.0001**	**<0.0001**
		FruHC	0.064	0.438	0.729		**<0.0001**	**<0.0001**
	#2	SaHF	**<0.0001**	**<0.0001**	**<0.0001**	**<0.0001**		**0.0004**
	#4	USaHF	**<0.0001**	**<0.0001**	**<0.0001**	**<0.0001**	**0.0004**	

Muscle			ND	StaHC	SucHC	FruHC	SaHF	USaHF
GF		ND		0.604	>0.99	0.834	0.700	0.594
		StaHC	0.604		>0.99	>0.99	>0.99	>0.99
		SucHC	>0.99	>0.99		>0.99	>0.99	>0.99
		FruHC	0.834	>0.99	>0.99		>0.99	>0.99
		SaHF	0.700	>0.99	>0.99	>0.99		>0.99
		USaHF	0.594	>0.99	>0.99	>0.99	>0.99	
FMT		ND		>0.99	0.703	0.248	>0.99	0.932
		StaHC	>0.99		>0.99	0.744	>0.99	>0.99
		SucHC	0.703	>0.99		>0.99	>0.99	>0.99
		FruHC	0.248	0.744	>0.99		0.917	>0.99
		SaHF	>0.99	>0.99	>0.99	0.917		>0.99
		USaHF	0.932	>0.99	>0.99	>0.99	>0.99	

BAT			ND	StaHC	SucHC	FruHC	SaHF	USaHF
GF		ND		0.612	0.985	0.970	**0.0005**	0.410
		StaHC	0.612		>0.99	>0.99	0.060	>0.99
		SucHC	0.985	>0.99		>0.99	**0.019**	0.975
		FruHC	0.970	>0.99	>0.99		**0.004**	0.940
	#7	SaHF	**0.0005**	0.060	**0.019**	**0.004**		0.549
		USaHF	0.410	>0.99	0.975	0.940	0.549	
FMT	#5	ND		**0.011**	**0.015**	>0.99	**<0.0001**	**<0.0001**
		StaHC	**0.011**		>0.99	**0.042**	0.080	0.056
		SucHC	**0.015**	>0.99		0.057	0.347	0.276
	#6	FruHC	>0.99	**0.042**	0.057		**<0.0001**	**<0.0001**
		SaHF	**<0.0001**	0.080	0.347	**<0.0001**		>0.99
		USaHF	**<0.0001**	0.056	0.276	**<0.0001**	>0.99	

eWAT			ND	StaHC	SucHC	FruHC	SaHF	USaHF
GF		ND		0.990	>0.99	>0.99	**<0.0001**	**<0.0001**
		StaHC	0.990		>0.99	>0.99	**<0.0001**	**<0.0001**
		SucHC	>0.99	>0.99		>0.99	**<0.0001**	**<0.0001**
		FruHC	>0.99	>0.99	>0.99		**<0.0001**	**<0.0001**
	#13	SaHF	**<0.0001**	**<0.0001**	**<0.0001**	**<0.0001**		**0.007**
	#15	USaHF	**<0.0001**	**<0.0001**	**<0.0001**	**<0.0001**	**0.007**	
FMT		ND		0.535	0.942	**0.027**	**<0.0001**	**0.001**
		StaHC	0.535		>0.99	0.867	**<0.0001**	**<0.0001**
		SucHC	0.942	>0.99		0.683	**<0.0001**	**<0.0001**
	#12	FruHC	**0.027**	0.867	0.683		**<0.0001**	**<0.0001**
	#14	SaHF	**<0.0001**	**<0.0001**	**<0.0001**	**<0.0001**		0.517
	#16	USaHF	**0.001**	**<0.0001**	**<0.0001**	**<0.0001**	0.517	

Liver			ND	StaHC	SucHC	FruHC	SaHF	USaHF
GF	#8	ND		>0.99	0.390	**0.020**	0.331	**0.024**
	#9	StaHC	>0.99		0.610	**0.032**	0.537	**0.043**
		SucHC	0.390	0.610		>0.99	>0.99	0.981
		FruHC	**0.020**	**0.032**	>0.99		>0.99	>0.99
		SaHF	0.331	0.537	>0.99	>0.99		>0.99
		USaHF	**0.024**	**0.043**	0.981	>0.99	>0.99	
FMT		ND		0.831	>0.99	>0.99	0.194	**<0.0001**
		StaHC	0.831		0.987	0.948	**0.0005**	**<0.0001**
		SucHC	>0.99	0.987		>0.99	0.062	**<0.0001**
		FruHC	>0.99	0.948	>0.99		**0.022**	**<0.0001**
	#10	SaHF	0.194	**0.0005**	0.062	**0.022**		**0.027**
	#11	USaHF	**<0.0001**	**<0.0001**	**<0.0001**	**<0.0001**	**0.027**	

Caecum			ND	StaHC	SucHC	FruHC	SaHF	USaHF
GF	#17	ND		**<0.0001**	**<0.0001**	**<0.0001**	**<0.0001**	**<0.0001**
	#18	StaHC	**<0.0001**		0.099	**0.030**	**0.001**	0.055
		SucHC	**<0.0001**	0.099		0.990	0.922	>0.99
		FruHC	**<0.0001**	**0.030**	0.990		0.852	>0.99
		SaHF	**<0.0001**	**0.001**	0.922	0.852		0.975
		USaHF	**<0.0001**	0.055	>0.99	>0.99	0.975	
FMT		ND		>0.99	0.989	0.986	0.981	>0.99
		StaHC	>0.99		>0.99	>0.99	>0.99	>0.99
		SucHC	0.989	>0.99		>0.99	>0.99	>0.99
		FruHC	0.986	>0.99	>0.99		>0.99	>0.99
		SaHF	0.981	>0.99	>0.99	>0.99		>0.99
		USaHF	>0.99	>0.99	>0.99	>0.99	>0.99	

SupplementaryTable S5. **Difference in microbiota abundance at phylum level between diet groups.**
Data indicate *p*-value analyzed by one-way analysis of variance and Tukey's test.
Bold letter, $p < 0.05$.

Bacteroidetes	ND	StaHC	SucHC	FruHC	SaHF	USaHF
ND		**<0.0001**	**<0.0001**	**<0.0001**	**<0.0001**	**<0.0001**
StaHC	**<0.0001**		0.981	0.065	0.949	**0.020**
SucHC	**<0.0001**	0.981		0.462	>0.99	0.167
FruHC	**<0.0001**	0.065	0.462		0.573	0.923
SaHF	**<0.0001**	0.949	>0.99	0.573		0.223
USaHF	**<0.0001**	**0.020**	0.167	0.923	0.223	

Actinobacteria	ND	StaHC	SucHC	FruHC	SaHF	USaHF
ND		0.835	>0.99	**<0.001**	0.984	>0.99
StaHC	0.835		0.935	**<0.001**	>0.99	0.930
SucHC	>0.99	0.935		**<0.001**	>0.99	>0.99
FruHC	**<0.001**	**<0.001**	**<0.001**		**0.002**	**<0.001**
SaHF	0.984	>0.99	>0.99	**0.002**		>0.99
USaHF	>0.99	0.930	>0.99	**<0.001**	>0.99	

Bacillota	ND	StaHC	SucHC	FruHC	SaHF	USaHF
ND		**<0.0001**	**<0.0001**	**0.007**	**<0.0001**	**<0.0001**
StaHC	**<0.0001**		0.932	**0.005**	>0.99	**0.013**
SucHC	**<0.0001**	0.932		0.152	0.976	**0.005**
FruHC	**0.007**	**0.005**	0.152		**0.027**	**<0.0001**
SaHF	**<0.0001**	>0.99	0.976	**0.027**		**0.026**
USaHF	**<0.0001**	**0.013**	**0.005**	**<0.0001**	**0.026**	

Tenericutes	ND	StaHC	SucHC	FruHC	SaHF	USaHF
ND		**0.004**	**0.012**	0.590	**0.020**	**0.014**
StaHC	**0.004**		>0.99	0.055	>0.99	>0.99
SucHC	**0.012**	>0.99		0.142	>0.99	>0.99
FruHC	0.590	0.055	0.142		0.223	0.159
SaHF	**0.020**	>0.99	>0.99	0.223		>0.99
USaHF	**0.014**	>0.99	>0.99	0.159	>0.99	

Verrucomicrobia	ND	StaHC	SucHC	FruHC	SaHF	USaHF
ND		0.900	>0.99	**<0.001**	>0.99	0.936
StaHC	0.900		>0.99	**<0.0001**	0.559	>0.99
SucHC	>0.99	>0.99		**<0.0001**	0.850	>0.99
FruHC	**<0.001**	**<0.0001**	**<0.0001**		**<0.001**	**<0.0001**
SaHF	>0.99	0.559	0.850	**<0.001**		0.673
USaHF	0.936	>0.99	>0.99	**<0.0001**	0.673	

Saccharibacteria	ND	StaHC	SucHC	FruHC	SaHF	USaHF
ND		0.239	0.848	0.106	0.189	0.189
StaHC	0.239		0.924	>0.99	>0.99	>0.99
SucHC	0.848	0.924		0.719	0.806	0.806
FruHC	0.106	>0.99	0.719		>0.99	>0.99
SaHF	0.189	>0.99	0.806	>0.99		>0.99
USaHF	0.189	>0.99	0.806	>0.99	>0.99	

Proteobacteria	ND	StaHC	SucHC	FruHC	SaHF	USaHF
ND		0.131	**<0.001**	0.509	0.869	0.495
StaHC	0.131		**0.032**	0.910	0.751	0.989
SucHC	**<0.001**	**0.032**		**0.004**	**0.005**	**0.022**
FruHC	0.509	0.910	**0.004**		0.996	>0.99
SaHF	0.869	0.751	**0.005**	0.996		0.984
USaHF	0.495	0.989	**0.022**	>0.99	0.984	

Fusobacteria	ND	StaHC	SucHC	FruHC	SaHF	USaHF
ND		0.521	0.180	0.653	0.247	0.180
StaHC	0.521		0.906	>0.99	0.962	0.906
SucHC	0.180	0.906		0.815	>0.99	>0.99
FruHC	0.653	>0.99	0.815		0.903	0.815
SaHF	0.247	0.962	>0.99	0.903		>0.99
USaHF	0.180	0.906	>0.99	0.815	>0.99	
